# Genome-wide association study identifies key loci and candidate genes for seed vigor in upland cotton (*Gossypium hirsutum* L.)

**DOI:** 10.3389/fpls.2026.1804577

**Published:** 2026-06-01

**Authors:** Huayuan Liu, Zhong Wang, Hongbing Sun, Ying Zou, Jingjing Ma, Shuaijun Wu, Sibanur Abdukerim, Kai Zheng, Quanjia Chen, Xiaojuan Deng

**Affiliations:** 1 College of Agronomy, Xinjiang Agricultural University, Urumqi, China; 2 Xinjiang Key Laboratory of Crop Biological Breeding, Urumqi, China; 3 Cotton Engineering Research Center of Ministry of Education, Urumqi, China

**Keywords:** candidate gene, genome-wide association study (GWAS), GO and KEGG enrichment analyzes, *Gossypium hirsutum* L., seed vigor

## Abstract

**Introduction:**

Rapid and uniformgermination of cotton seeds is a prerequisite for achieving high and stable yields. Elucidating the molecular mechanisms underlying seed germination will help establish a genetic foundation for improving seed vigor, which is a key agronomic trait.

**Methods:**

In this study, seeds from 259 *Gossypium hirsutum* germplasm accessions grown in seven environments over two years were evaluated for eight seed-vigor-related traits, including germination potential, germination rate, germination index, and vigor index. These eight traits exhibited abundant phenotypic variation within the population and were strongly correlated with each other. Correlation analysis, principal component analysis, and random forest importance evaluation identified the germination index, germination potential, and vigor index as the three traits most significantly and closely associated with seed vigor. Therefore, these three traits were used for genome-wide association analysis to identify important loci and genes controlling seed germination vigor in cotton.

**Results and discussion:**

Using a mixed linear model (MLM), six stable SNPs associated with the germination index (GI), three with germination potential (GP), and six with vigor index (VI) were identified, yielding a total of 15 stably associated SNPs across the three traits in multiple environments. Among them, locus A09:47545163 was repeatedly detected for both the germination index and potential. Using a 500-kb interval as the reference distance for candidate region delineation, 166 candidate genes harboring nonsynonymous mutations were identified. Further integrating GO/KEGG enrichment analysis, genotype effects at significant loci, and gene functional annotation, we prioritized 10 candidate genes that are potentially involved in the formation and regulation of seed vigor in cotton. Meanwhile, public TM-1 transcriptomic expression data provided exploratory supporting evidence for the functional inference of some candidate genes. These findings provide new evidence for elucidating the genetic basis of seed vigor in upland cotton and offer valuable gene resources for the development of high-vigor germplasm and related molecular markers.

## 1 Introduction

Seed germination marks the beginning of the plant life cycle and represents the earliest developmental stage at which plants perceive external environmental cues. Seed vigor reflects the potential of seeds to germinate rapidly and uniformly and establish normal seedlings under favorable or complex environmental conditions. It is directly associated with field emergence uniformity, seedling establishment quality, and final yield formation (Wang et al., 2025). Cotton is an important economic crop and the primary natural fiber source in the textile industry. *Gossypium hirsutum* is the most widely cultivated cotton species in agricultural production (Chen et al., 2007; Qian et al., 2020). With the advancement of mechanized precision sowing, the requirements for rapid and uniform germination in cotton have become increasingly stringent. Therefore, elucidating the genetic basis underlying cotton seed vigor and identifying the genes involved are of great significance for achieving high and stable yields and for cotton cultivar improvement. In most crops, the life cycle begins with seed germination and ends with seed production, during which seed germination vigor serves as an important agronomic trait that directly or indirectly affects crop yield and quality (Li et al., 2022). Previous studies have demonstrated that seed vigor is a complex quantitative trait jointly influenced by genetic and environmental factors, and its establishment involves multiple biological processes, including dormancy release, resumption of embryo growth, mobilization of storage reserves, and stress response. Among these, the dynamic balance between abscisic acid (ABA) and gibberellin (GA) is considered one of the core mechanisms regulating seed germination, whereas reactive oxygen species (ROS) signaling also plays an important role in germination initiation and environmental responses (Gong et al., 2022; Zhao et al., 2022). In recent years, with the development of high-throughput sequencing technologies and statistical genetics, genome-wide association studies (GWAS) have become an important approach for dissecting the genetic basis of complex traits. Multiple QTLs and candidate genes associated with seed germination and vigor have been identified in crops such as rice, Arabidopsis thaliana, and maize. These studies have provided an important foundation for elucidating the molecular regulatory mechanisms of seed vigor and have also demonstrated that GWAS is an effective strategy for identifying genetic loci and candidate genes associated with seed vigor (Shi et al., 2017; Ma et al., 2022; Liu et al., 2024). In contrast, genetic studies on seed vigor in cotton are relatively limited. Existing studies have shown that GWAS has been widely applied in cotton for the genetic dissection of disease resistance, stress tolerance, and fiber quality traits (Thyssen et al., 2019; Song et al., 2021; Mohammed et al., 2024; Wang et al., 2025; Wei et al., 2025). With respect to seed germination and vigor, a few studies have identified loci and candidate genes associated with seed germination or seed vigor in *Gossypium hirsutum (*Si et al., 2022; Li et al., 2023). However, current cotton-related studies have focused mainly on germination or seedling tolerance under stress conditions, such as salinity, low phosphorus, and low temperature (Sun et al., 2018; Shen et al., 2023). Systematic investigations into the genetic architecture, stable association loci, and candidate genes underlying seed vigor under normal germination conditions in *Gossypium hirsutum* are still lacking. Therefore, the key genetic loci that regulate seed vigor in *Gossypium hirsutum*, the phenotypic indices that best characterize seed vigor, and the genes that may participate in its molecular regulation remain scientific questions worthy of further investigation. Based on this background, the present study used 259 *Gossypium hirsutum* germplasm accessions as materials and integrated seed-vigor phenotypic data collected across seven environments over two years to systematically evaluate the variation patterns and stability of seed-vigor-related traits, identify key indices that can effectively characterize seed vigor, and subsequently perform genome-wide association analysis. Furthermore, in this study, candidate genes were comprehensively prioritized by integrating multi-environment stable association signals, candidate interval consolidation, genotype effects at significant loci, functional annotation of candidate genes, and GO/KEGG enrichment analysis. Public TM-1 expression profile data were also used as exploratory supporting evidence to provide candidate gene resources for subsequent functional validation. The results of this study will provide a theoretical basis for a deeper understanding of the molecular regulatory mechanisms underlying cotton seed vigor, as well as for the identification and utilization of high-vigor germplasm resources.

## 2 Materials and methods

### 2.1 Plant materials and field experiments

A natural population comprising 259 upland cotton (*Gossypium hirsutum* L.) accessions was used. The accessions originated from major cotton-producing regions, including the Yellow River Basin, Yangtze River Basin, and Northwest Inland Region, as well as other production areas in China and international sources (**Supplementary Table 1**). All materials were provided by the Xinjiang Key Laboratory of Crop Breeding, Xinjiang Agricultural University (Urumqi, China). In 2023, field trials were conducted at three sites in Xinjiang, China. The sites included Yuepuhu County, Kashgari Prefecture in southern Xinjiang (23YPH, 39°15′N, 76°48′E), Wensu County, Aksu Prefecture in southern Xinjiang (23WS, 41°18′N, 80°26′E), and the Liuhudi experimental base in Manas County, Changji Prefecture in northern Xinjiang (23LHD, 44°39′N, 86°08′E). In 2024, trials were conducted at four different sites. These included 24YPH, 24WS, the experimental base in Kuitun City, Ili Prefecture in northern Xinjiang (24KT, 44°26′N, 84°56′E), and the Sanping experimental base at the Lugang Campus of Xinjiang Agricultural University in Urumqi, northern Xinjiang (24SP, 43°56′N, 87°21′E). Sowing was performed from mid-to-late April, and harvesting was performed from mid-to-late October in both years. The experiments were conducted using a randomized complete-block design.

### 2.2 Phenotypic evaluation of seed vigor-related traits

After reaching conventional harvest maturity in each environment, seeds from each accession were harvested and subjected to an indoor germination assay. Following harvest, the cotton seeds were ginned, delinted with sulfuric acid, and thoroughly air-dried. For each accession, 60 seeds of uniform size were selected, surface-sterilized in 5% sodium hypochlorite solution for 10 min, and rinsed thoroughly with distilled water. The treated seeds were placed in sterilized Petri dishes lined with two layers of sterile filter paper, to which 10 mL of distilled water was added to ensure that the seeds were evenly distributed and laid flat. The germination test was conducted in an illuminated growth chamber under controlled conditions of 25 °C with a 16 h light/8 h dark photoperiod. Three biological replicates were set for each accession, with 20 seeds per replicate. Germination was recorded daily from 24 h after incubation for seven consecutive days. Seeds were considered normally germinated when the radicle emerged through the seed coat and the seedling met the criteria for normal germination. Germination potential was recorded on day 3, and the germination rate was recorded on day 7. On day 7, five normal seedlings were randomly selected from each replicate to measure the primary root length, seedling length, and fresh weight. The seedlings were then heat-treated at 105 °C for 30 min, followed by oven-drying at 80 °C to a constant weight for dry weight determination. Based on the germination process and seedling growth performance, eight seed-vigor-related traits were evaluated: germination potential (GP), germination rate (GR), germination index (GI), vigor index (VI), primary root length (RL), seedling length (OL), fresh weight (SW), and dry weight (DW).

The formulas used for trait calculations are as follows:

Germination potential (GP, %):


$$\text{GP}=\frac{\text{Number of normally germinated seeds on day }3}{\text{Total number of tested seeds}}\times 100$$

Germination rate (GR, %):


$$\text{GR}=\frac{\text{number of normally germinated seeds on day }7}{\text{total number of tested seeds}}\times 100$$

Germination index (GI):


$$\text{GI}=\sum \frac{{\text{G}}_{\text{t}}}{{\text{D}}_{\text{t}}}$$

Vigor index (VI):


VI=GI×S

Where G_t_ is the number of seeds germinated on day t, D_t_ is the corresponding day of germination, and S is the mean fresh weight per normal seedling (g) based on seedlings randomly sampled on day 7.

### 2.3 Statistical analysis of seed-vigor phenotypic data, analysis of variance, and heritability estimation

Phenotypic data collected across seven environments over two years were first analyzed using SPSS software to obtain descriptive statistics, including the maximum, minimum, mean, and standard deviation for each trait. Subsequent statistical analyzes were performed using R (version 4.5.2). The FactoMineR package was used for correlation analysis and principal component analysis (PCA) (Yuan et al., 2025), and the randomForest package was used to evaluate variable importance in the random forest model (Zhang et al., 2024) to identify key phenotypic traits for subsequent genome-wide association study (GWAS).

To evaluate the genetic stability and environmental response patterns of each trait under multi-environment conditions, a mixed linear model was used to perform a combined analysis of variance for the eight seed-vigor-related traits. The combined analysis model was as follows:


$$y_{ijk}=\mu +E_{j}+G_{i}+{\left(GE\right)}_{ij}+R_{k(j)}+\epsilon_{ijk}$$

Where *y_ijk_* is the observed value of the *j*th genotype in the *i*th environment and the *k*h replicate; *μ* is the overall mean; *E_j_* is the effect of the *i*th environment; *G_i_* is the effect of the *j*th genotype; (*GE*)*_ij_* is the genotype-by-environment interaction effect; *R_k_*_(_*_j_*_)_ is the replicate effect nested within environment; and *ϵ_ijk_* is the random residual error. The broad-sense heritability across environments was estimated as follows:


$$H^{2}=\frac{\sigma_{G}^{2}}{\sigma_{G}^{2}+\sigma_{\mathrm{GE}}^{2}/e+\sigma_{e}^{2}/\left(e\times r\right)}$$

Where e is the number of environments and i is the number of replicates.

In addition, the lme4 package was used to calculate the best linear unbiased estimators (BLUEs) for each trait in the seven individual environments (23YPH, 23WS, 23LHD, 24YPH, 24WS, 24KT, and 24SP) and across-environment combined model. These BLUE values were subsequently used for correlation analysis, principal component analysis, random forest evaluation and downstream GWAS.

### 2.4 Whole-genome resequencing of the study panel

In a previous study conducted by our research group, 314 cotton accessions were subjected to whole-genome resequencing using the Illumina HiSeq 2500 platform (Deng., 2022). Based on this dataset, 259 *Gossypium hirsutum* germplasm accessions were selected in the present study for subsequent analyzes. The average sequencing depth was 10× and the reference genome coverage reached 90%. The reference genome used was the TM-1 (CRI) assembly. Raw SNP data were subjected to quality control using the PLINK software (Purcell et al., 2007). Among the initial 6,607,760 SNPs, markers were filtered according to a minor allele frequency (MAF) ≥ 0.05 and a missing rate< 0.05, resulting in a final set of 1,144,681 high-quality SNPs.

### 2.5 Population structure and linkage disequilibrium analysis

To evaluate phylogenetic relationships at the whole-genome level, a phylogenetic tree was constructed using the neighbor-joining (NJ) method implemented in the TASSEL software. Population structure was inferred using ADMIXTURE, with the number of clusters (*K*) ranging from 2 to 9 and 10,000 iterations performed for each run. Principal component analysis (PCA) was conducted using GCTA to further assess population stratification. Linkage disequilibrium (LD) was estimated by calculating pairwise LD coefficients (r^2^) among high-quality SNPs, and LD decay patterns were analyzed using PopLDdecay.

### 2.6 Genome-wide association analysis of key seed-vigor traits

Based on the calculated BLUE values of the phenotypic traits, a genome-wide association analysis (GWAS) was performed using the mixed linear model implemented in GEMMA. The GWAS results were visualized as Manhattan and quantile–quantile (Q–Q) plots using the CMplot package in R version 4.5.2. The significance threshold was defined according to the number of effectively independent SNPs (N)). Independent markers were determined using PLINK with a sliding-window approach (window size = 50, step size = 10, and r^2^ threshold = 0.01), yielding N = 21,062. The suggested threshold (1/N) was then calculated and used as the significance threshold accordingly and –log_10_(p) = 4.323 was adopted as the cutoff for subsequent analyzes in order to identify a larger set of reliable candidate genes.

### 2.7 Phenotypic variation analysis among different genotypes at significant SNP loci

To visually illustrate phenotypic differences among different genotypes at significant SNP loci across environments, the phenotypic values of accessions carrying different genotypes were compared under single-environment conditions. According to the distribution of genotype samples within each environment, the accessions were classified into different genotype groups. Genotype groups represented by no fewer than three accessions in a given environment were included in subsequent statistical analyzes. For data meeting the sample size requirement, one-way analysis of variance (ANOVA) was performed, followed by Tukey’s honestly significant difference (HSD) test for multiple comparisons among groups, and the results were annotated using significance asterisks. This analysis was mainly used for visual presentation and auxiliary comparison of phenotypic differences among candidate significant loci, and its results were intended to support the intuitive display of candidate locus effects rather than serve as the sole basis for rigorous association inference.

### 2.8 Candidate gene prediction

Based on the significant SNPs identified by genome-wide association analysis (GWAS), the SNPs were first sorted according to their physical positions on chromosomes. With reference to the linkage disequilibrium (LD) decay distance estimated for the study population and previous related reports, a 500-kb interval was used as the reference distance for defining candidate regions (Dong et al., 2018). When the physical distance between adjacent significant SNPs was less than or equal to 500 kb, they were merged into the same candidate interval; when the physical distance exceeded 500 kb, they were assigned to different candidate intervals. Based on these candidate intervals, SnpEff was used to annotate the functional effects of sequence variants within each interval, and genes harboring nonsynonymous mutations were retained as the candidate genes. Further comprehensive functional annotation of genes within the candidate intervals was performed by integrating the functional annotation information of the TM-1 reference genome of Gossypium hirsutum from the CottonMD database (Yang et al., 2023) with the functional descriptions of homologous genes available from the TAIR resource for Arabidopsis thaliana. In addition, the 166 genes carrying nonsynonymous mutations were subjected to GO and KEGG enrichment analyzes using the OmicStudio platform of Majorbio Cloud, with genes in the reference genome with corresponding functional annotations used as the background set. The enrichment P and Q values for each GO term and KEGG pathway were calculated, and the top significantly enriched GO terms and KEGG pathways were selected for visualization. The candidate genes were then comprehensively prioritized to provide a basis for the subsequent identification of key candidate genes.

## 3 Results

### 3.1 Phenotypic variation of seed vigor-related traits in upland cotton

**Figure 1:**
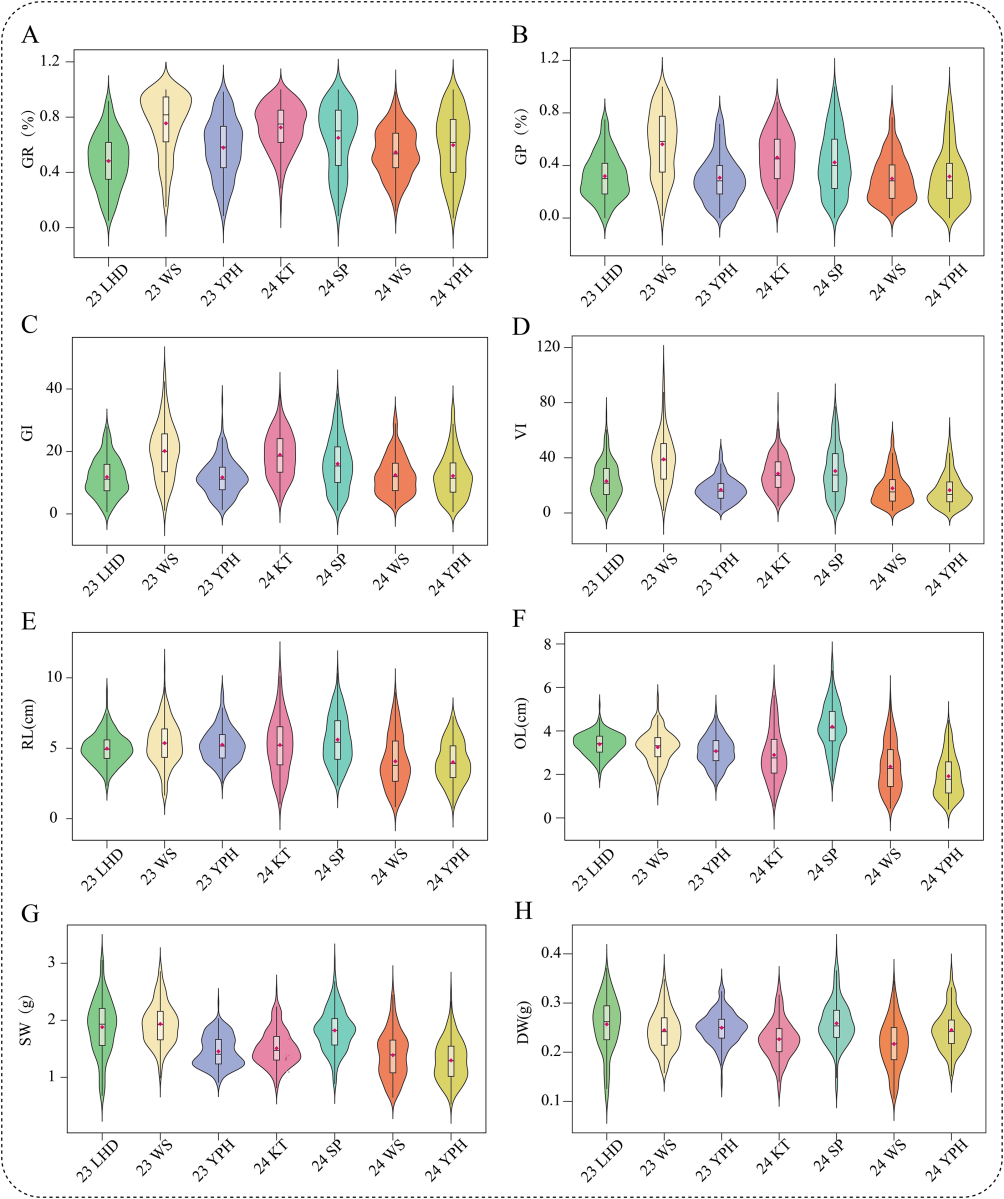
Violin plots of eight vigor-related traits across seven environments in *G. hirsutum*. **(A)** GR, germination rate; **(B)** GP, germination potential; **(C)** GI, germination index; **(D)** VI, vigor index; **(E)** RL, root length; **(F)** OL, shoot length; **(G)** SW, fresh weight; **(H)** DW, dry weight. 23LHD: 2023 Liuhudi; 23WS: 2023 Wensu; 23YPH: 2023 Yuepuhu; 24KT: 2024 Kuitun; 24SP: 2024 Sanping; 24WS: 2024 Wensu; 24YPH: 2024 Yuepuhu.

Descriptive statistics were generated for the eight seed vigor-related traits evaluated across the seven environments in 2023 and 2024 (**Supplementary Table 2**). Substantial phenotypic variation was detected for all traits in the association panel, including germination potential, germination rate, germination index, vigor index, root length, shoot length, fresh weight, and dry weight. The vigor index exhibited the largest coefficient of variation (CV) at 59.31%, followed by germination potential, with a coefficient of variation of 57.49%. Dry weight had the lowest coefficient of variation (20.18%). The kurtosis and skewness values for all traits ranged from -1.03 to 1.07, respectively. Consistent with the violin plot distributions across the seven environments (**Figure 1**), these results indicate that trait values approximated a normal distribution under the tested conditions. Collectively, these distributions support the suitability of this population for downstream GWAS.

### 3.2 Correlation and principal component analysis of seed vigor-related traits in upland cotton

**Figure 2:**
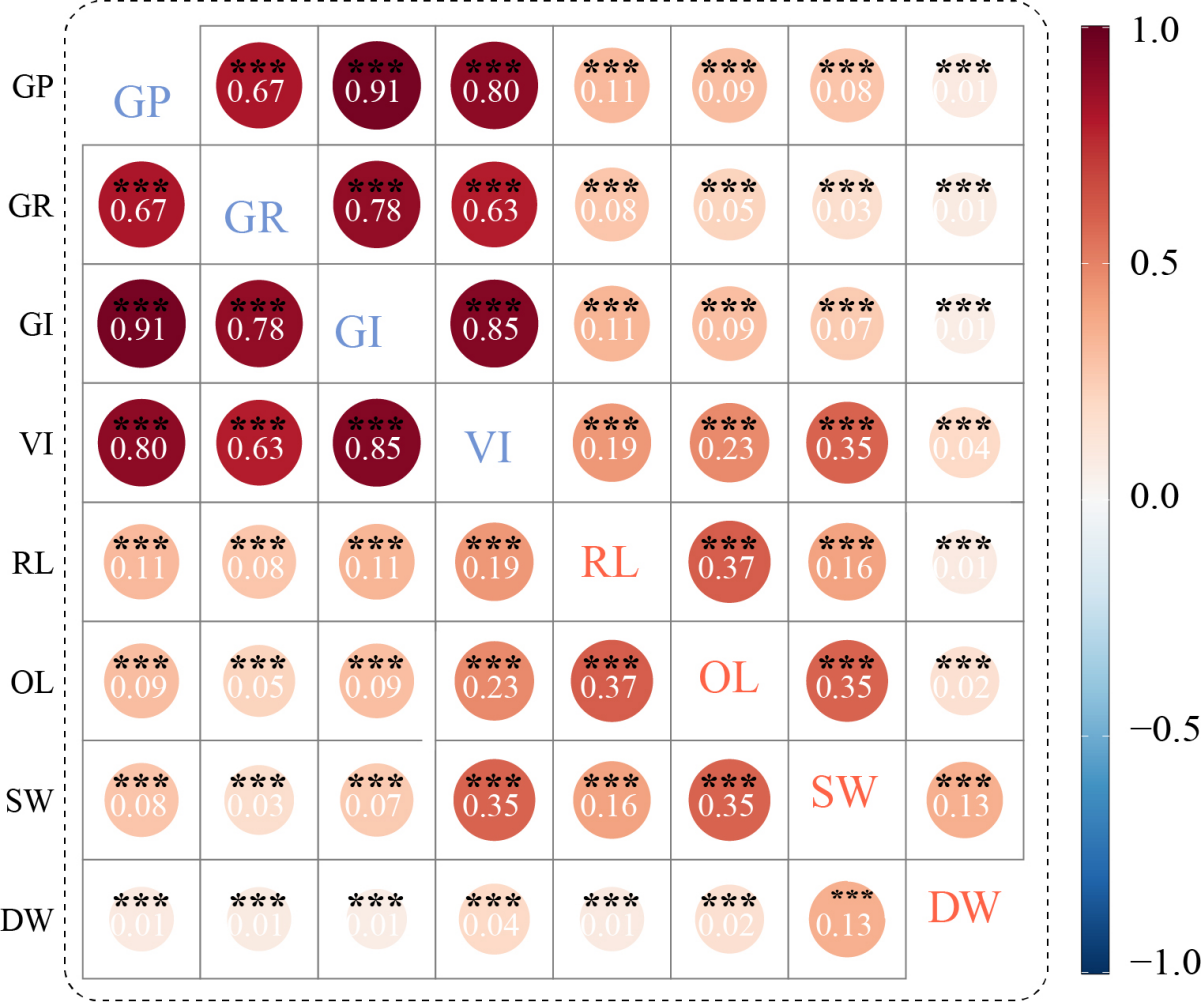
Correlation matrix of the eight seed vigor-related traits. GR, germination rate; GP, germination potential; GI, germination index; VI, vigor index; RL, root length; OL, shoot length; SW, fresh weight; DW, dry weight. Statistical significance levels are indicated as follows: *p<0.05, **p<0.01, ***p<0.001.

**Figure 3:**
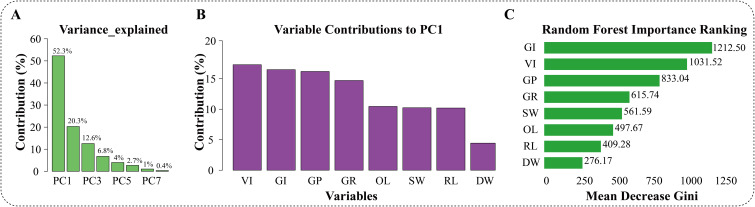
Principal component analysis of the eight seed vigor-related traits of G. hirsutum. **(A)** Variance explained by each principal component. **(B)** Contribution of the eight traits to PC1. **(C)** Ranking of trait importance based on the Gini index from the random forest model. GR, germination rate; GP, germination potential; GI, germination index; VI, vigor index; RL, root length; OL, shoot length; SW, fresh weight; DW, dry weight.

Correlation and principal component analyzes (PCA) were conducted for eight seed vigor-related traits across 259 accessions. Correlation analysis revealed significant positive associations among all traits, with all pairwise correlations reaching statistical significance at P< 0.01 (**Figure 2**). The strongest correlation was observed between the germination index and germination potential (r = 0.91), followed by the association between the vigor index and GI (r = 0.85). The correlation between VI and GP was also strong (r = 0.80). The PCA indicated that the proportion of variance explained by the individual components ranged from 52.3% to 0.4% (**Figure 3A**). The first principal component (PC1) accounted for the greatest proportion of the variation (52.3%). Trait loading analysis revealed that VI, GI, and GP were the primary contributors to PC1 (**Figure 3B**). Consistently, random forest analysis based on the Gini importance ranking identified GI, GP, and VI as the three most informative traits (**Figure 3C**).

### 3.3 Analysis of variance and heritability of seed vigor-related traits in upland cotton

Based on phenotypic data collected from a natural population of 259 upland cotton accessions across seven environments over two years, a multi-environment analysis of variance was conducted for three seed vigor-related traits: germination potential (GP), germination index (GI), and vigor index (VI) (**Supplementary Table 3**). The results showed that all three traits were jointly affected by genotype (G), environment (E), and genotype × environment interaction (G×E). The genotypic effect was highly significant for GP, GI, and VI (P< 0.001). Meanwhile, the mean squares and F-values for both the environmental effect and the (G×E) interaction were also high, indicating that seed vigor-related traits in cotton are typical quantitative traits jointly regulated by genetic and environmental factors, with strong environmental sensitivity. Broad-sense heritability H^2^ was further estimated using a mixed linear model. The H^2^ values for GP, GI, and VI were 64.68%, 64.21%, and 50.15%, respectively. Among them, GP and GI exhibited relatively high genetic stability, whereas VI showed moderately high heritability. Taken together, these results indicate that although seed vigor-related traits are readily influenced by environmental conditions and (G×E) interactions, GI, GP, and VI still maintain relatively good genetic stability across multiple environments. This finding is consistent with previous studies on seed germination and seedling traits in upland cotton (Si et al., 2022; Li et al., 2023). Therefore, in combination with principal component analysis, random forest importance evaluation, and multi-environment broad-sense heritability analysis, GI, GP, and VI were selected as the key traits for GWAS.

### 3.4 Population structure and linkage disequilibrium analysis

**Figure 4:**
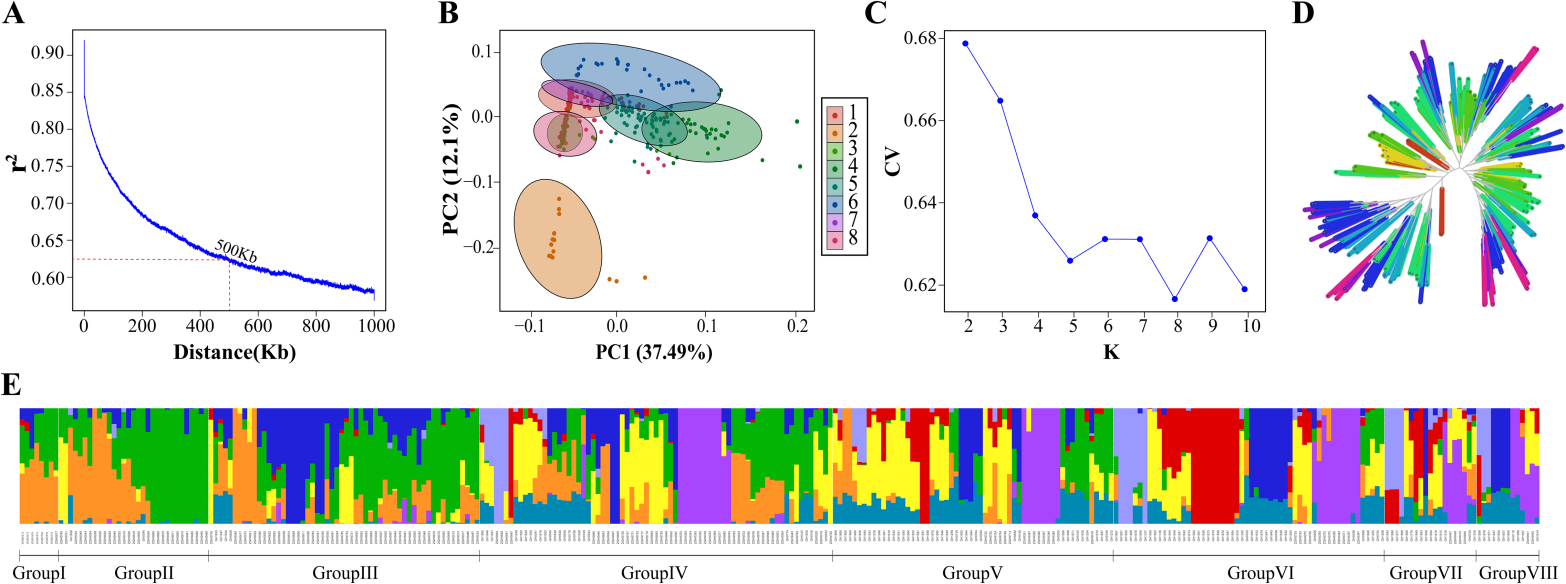
Population genetic analyzes of 259 upland cotton accessions. Note: **(a)** Genome-wide linkage disequilibrium (LD) decay. **(b)** Principal component analysis (PCA). **(c)** Cross-validation error used to determine the optimal number of subpopulations (K = 8). **(d)** Phylogenetic tree based on the K = 8 model. **(e)** Population structure inferred at K = 8.

Based on the ADMIXTURE analysis of population structure, the cross-validation results supported K = 8 as the optimal number of subpopulations (**Figure 4C**). This grouping pattern was consistent with the results of the neighbor-joining (NJ) phylogenetic tree and principal component analysis (PCA) (**Figures 4B, D**), providing evidence of clear genetic differentiation within this natural population. Genome-wide LD decay analysis further showed that the linkage disequilibrium coefficient (r^2^) reached a plateau at approximately 500 kb (**Figure 4A**), indicating that the SNP marker density used in this study was sufficient to meet the mapping resolution requirements for the subsequent GWAS.

### 3.5 Genome-wide association study of key seed vigor traits in upland cotton

**Figure 5:**
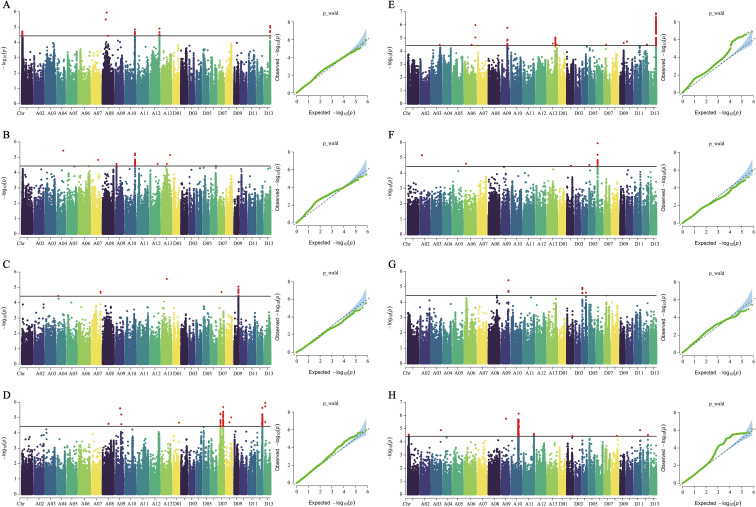
Manhattan plots from the genome-wide association study (GWAS) for the germination index (GI) in upland cotton. The integrated analysis across all seven environments (all-7Env) is shown in **(A)**, while panels **(B–D)** present results from the three trial sites in 2023: 23LHD, Liuhudi; 23WS, Wensu; and 23YPH, Yuepuhu. Results from the four trial sites in 2024 are displayed in panels **(E–H)**: 24KT, Kuitun; 24SP, Sanping; 24WS, Wensu; and 24YPH, Yuepuhu.

**Figure 6:**
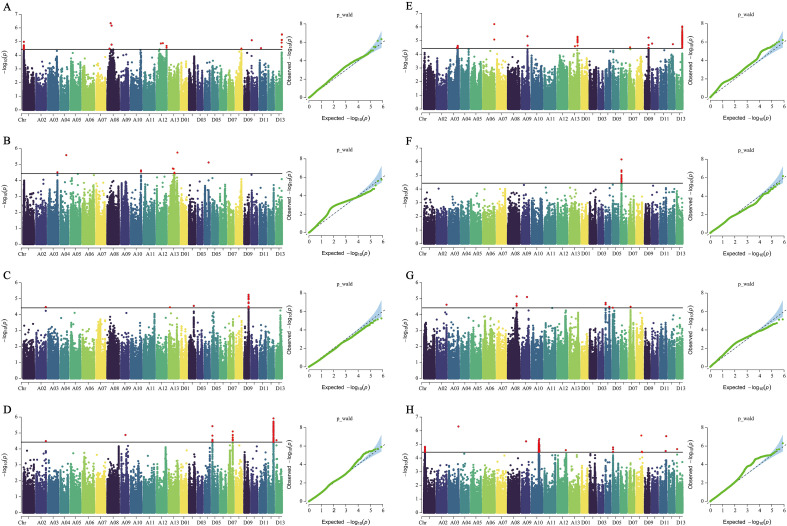
Manhattan plots of genome-wide association study (GWAS) for germination potential (GP) in upland cotton. **(A):** shows the integrated analysis across all seven environments (All-7Env), while panels **(B–D)** present results from the three trial sites in 2023; 23LHD, Liuhudi; 23WS, Wensu; and 23YPH, Yuepuhu. Results from the four trial sites in 2024 are displayed in panels **(E–H)**: 24KT, Kuitun; 24SP, Sanping; 24WS, Wensu, and 24YPH, Yuepuhu.

**Figure 7:**
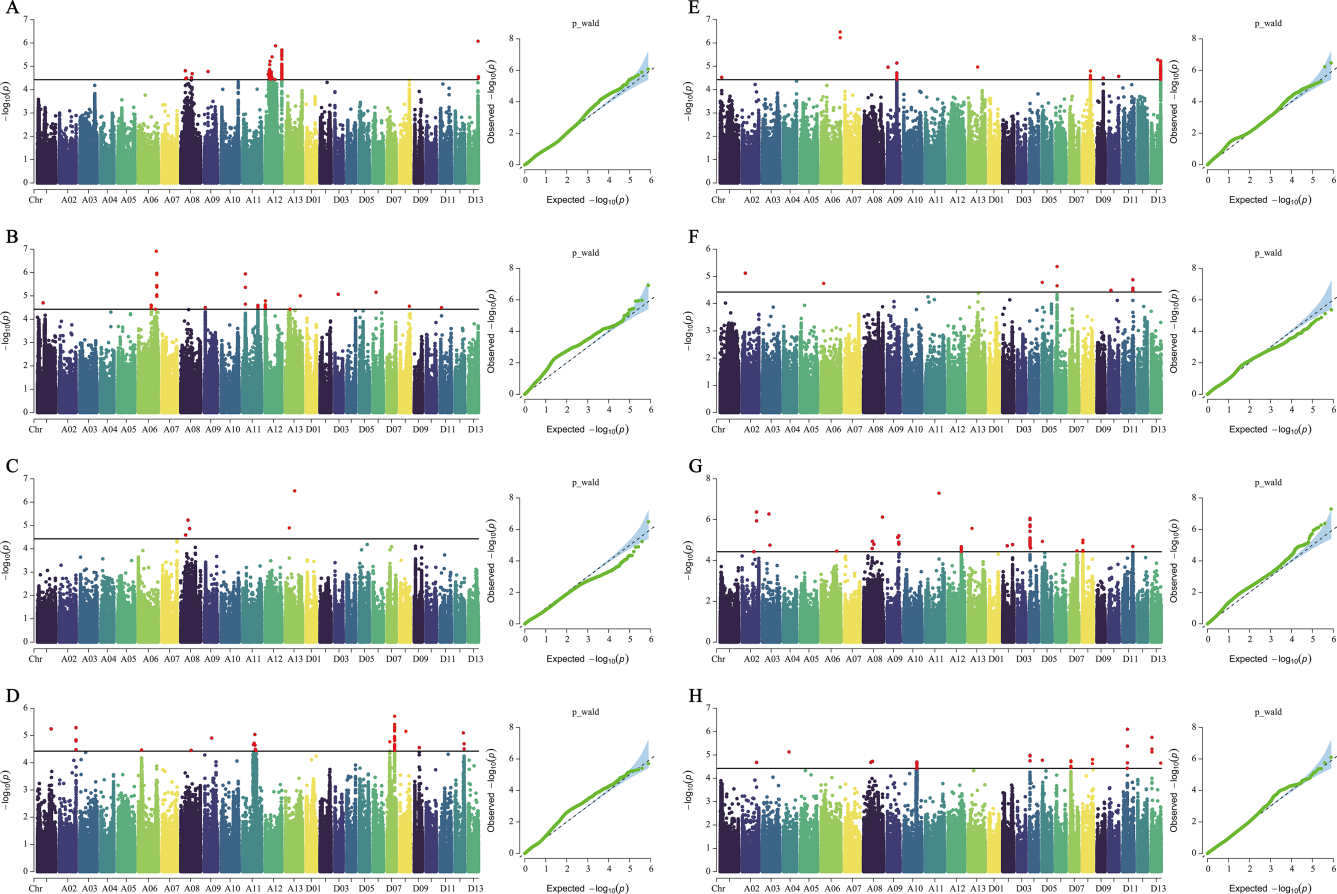
Manhattan plots of genome-wide association study (GWAS) for Vigor index (VI) in upland cotton. The integrated analysis across all seven environments (all-7Env) is shown in **(A)**, while panels **(B–D)** present results from the three trial sites in 2023: 23LHD, Liuhudi; 23WS, Wensu, and 23YPH, Yuepuhu. Results from the four trial sites in 2024 are displayed in panels **(E–H)**: 24KT, Kuitun; 24SP, Sanping; 24WS, Wensu; and 24YPH, Yuepuhu.

Based on the best linear unbiased estimators (BLUEs) of the phenotypic traits, genome-wide association analysis (GWAS) of seed vigor and SNP markers was performed using the mixed linear model (MLM) implemented in GEMMA. Eight association analysis datasets were included, comprising seven single-location field environments and the Overall BLUE across all seven environments (Overall BLUE across seven environments: 23LHD, Liuhudi in 2023; 23YPH, Yuepuhu in 2023; 23WS, Wensu in 2023; 24YPH, Yuepuhu in 2024; 24WS, Wensu in 2024; 24KT, Kuitun in 2024; and 24SP, Sanping in 2024). Using –log10 (P) ≥ 4.323 as the significance threshold, a total of 15 SNP loci showing significant associations in at least two environments were identified. These loci were distributed across seven chromosomes: A09, A10, D05, D13, A02, A08, and D04. For germination index (GI), 613 SNPs were detected across the eight environments (**Supplementary Table 4**). Among them, six SNP loci were significantly associated in at least two environments, all of which were located on chromosomes A09 and A10, with phenotypic variation explained ranging from 5.64% to 9.34% (**Figure 5**; **Table 1**). For germination potential (GP), 454 SNPs were detected across the eight environments (**Supplementary Table 5**). Among these, three SNP loci were significantly associated in at least two environments, distributed on chromosomes A09, D05, and D13, and explained 7.09% to 8.59% of the phenotypic variation (**Figure 6**; **Table 1**). Notably, SNP locus A09:47545163 was detected in both GI and GP. For the vigor index (VI), 337 SNPs were identified across the eight environments (**Supplementary Table 6**). Of these, six SNP loci showed significant associations in at least two environments and were located on chromosomes A02, A08, D04, and D13, explaining 7.42% to 9.78% of the phenotypic variation (**Figure 7**; **Table 1**).

**Table 1:** Summary of combined association intervals of 15 significant SNPs for the three traits.

Trait	Chr	Position	SNP	Env	Chromosome interval	-log10 (*p*)	Pve (%)
GI	A10	109096218	A10_109096218	23LHD、All-7Env	A10:108596217–109624660 73 genes	4.59-4.70	5.73–7.29
	A10	109120366	A10_109120366	23LHD、All-7Env		4.51-4.69	5.86–6.96
	A10	109120427	A10_109120427	23LHD、All-7Env		4.84-5.24	6.07–8.20
	A10	109124473	A10_109124473	23LHD、All-7Env		4.53-4.64	5.64–7.19
	A10	109124660	A10_109124660	23LHD、All-7Env		4.54-4.78	5.65–7.43
	A09	47545163	A09_47545163	23YPH、24YPH	A09:47045162–48045163 21 genes	5.60-5.76	8.88–9.34
GP	A09	47545163	A09_47545163	23YPH、24YPH		4.88-5.76	7.65–8.41
	D05	4875896	D05_4875896	23YPH、24YPH	D05:4375895–5375896 135 genes	4.64-5.43	7.39–8.59
	D13	5100227	D13_5100227	23YPH、24YPH	D13:4600226–5600227 70 genes	4.55-4.65	7.09–7.42
VI	A02	91069897	A02_91069897	24YPH、24WS	A02:90569896–91569897 3 genes	4.68-5.94	7.47–9.57
	A08	56855259	A08_56855259	24YPH、24WS	A08:56355258–57355259 4 genes	4.73-4.93	7.55–7.85
	D04	3372587	D04_3372587	24YPH、24WS	D04:2872586–3874750 76 genes	4.96-6.00	7.96–9.67
	D04	3373713	D04_3373713	24YPH、24WS		4.98-6.06	8.00–9.78
	D04	3374750	D04_3374750	24YPH、24WS		4.75-5.43	7.59–8.70
	D13	60741776	D13_60741776	24YPH、All-7Env	D13:60241775–61241776 67 genes	4.65-6.08	7.42–7.81

Linkage disequilibrium (LD) analysis was further performed for the 15 SNPs significantly associated with seed vigor in Gossypium hirsutum, and candidate intervals were merged within 500 kb upstream and downstream of each significant SNP. When the physical distance between adjacent significant SNPs was less than or equal to 500 kb, they were merged into the same candidate interval; when the distance exceeded 500 kb, they were assigned to different candidate intervals. For the three traits, GI, GP, and VI, the 15 significant SNPs were ultimately consolidated into eight candidate intervals containing a total of 449 genes, including 94 genes for GI, 205 genes for GP, and 150 genes for VI. In addition, the overlapping locus A09:47545163, shared by GI and GP, contained 21 genes (**Supplementary Table 7**).

### 3.6 Phenotypic variation analysis among different genotypes at significant SNP loci

To further validate the association effects of significant SNP loci on seed-vigor-related traits, comparative analyzes of genotype-dependent phenotypic variation were performed for three major traits, namely the germination index (GI), germination potential (GP), and vigor index (VI), at different significant loci (**Table 2**; **Supplementary Figure S8**). The results indicated that genotypic effects varied to some extent among loci associated with different traits.

**Table 2:** Summary of phenotypes corresponding to different genotypes of the 15 significant SNPs.

Trait	SNP	Ref	Alt	Ref/Ref	Alt/Alt	Ref/Alt	Missing	N
GI	A09:47545163	A	T	220	0	27	12	259
	A10:109096218	G	A	217	27	0	15	259
	A10:109120366	G	A	219	28	0	12	259
	A10:109120427	T	A	219	28	0	12	259
	A10:109124473	A	G	225	29	1	4	259
	A10:109124660	A	G	223	29	0	7	259
GP	A09:47545163	A	T	220	0	27	12	259
	D05:4875896	G	T	87	156	5	11	259
	D13:5100227	A	C	233	13	4	9	259
VI	A02:91069897	T	C	3	1	255	0	259
	A08:56855259	T	C	30	215	6	8	259
	D04:3372587	C	T	242	14	1	2	259
	D04:3373713	C	T	241	14	1	3	259
	D04:3374750	T	C	235	14	1	9	259
	D13:60741776	G	T	41	17	201	0	259

Based on the integrated analysis of genotype sample size distribution, magnitude of phenotypic differences across environments, and results of significance tests, locus A09:47545163 exhibited the most stable genotypic effect for GI and GP. At this locus, 220 accessions carried the AA genotype and 27 accessions carried the AT genotype, whereas no TT genotype was detected. Nevertheless, the violin plots across environments consistently showed that accessions with the AT genotype generally displayed higher GI and GP values than those with the AA genotype in most environments, and the differences reached significant or highly significant levels in some environments. These results suggest that the heterozygous genotype at this locus exerts a pronounced positive effect on germination index and germination potential, indicating that the AT genotype may be favorable for GI and GP. For the vigor index (VI), the clearest phenotypic separation was observed at locus D13:60741776, where all three genotypes were represented by a reasonable number of accessions, with 41, 201, and 17 accessions carrying the GG, GT, and TT genotypes, respectively, thus providing a relatively high reliability for the comparison. The phenotypic distributions across environments showed that in most environments, accessions with the GG genotype had the highest VI values, whereas those with the TT genotype tended to have the lowest values, with the GT genotype generally intermediate between the two. Significant differences were detected in multiple environments, indicating that the homozygous reference allele genotype at this locus is more favorable for the maintenance and improvement of the vigor index. Therefore, the GG genotype may be considered a favorable genotype for VI.

In summary, the phenotypic variation analysis among different genotypes at significant SNP loci showed that locus A09:47545163 exhibited a relatively stable positive effect on GI and GP across multiple environments, with the AT genotype likely representing a favorable genotype. For VI, locus D13:60741776 showed the clearest genotypic effect, and the GG genotype was likely to be favorable. These findings indicate that the genetic regulation of different seed-vigor traits shares certain common features while also exhibiting clear locus specificity, thereby providing useful evidence for subsequent candidate-gene screening, identification of superior allelic variants, and marker-assisted selection. .

### 3.7 Candidate gene prediction

Based on the physical positions of the 15 multi-environment stable associated SNPs and the 500-kb criterion for candidate interval definition, a total of eight merged associated intervals were identified in this study, encompassing 449 genes. Among them, the GI-related intervals contained 94 genes, the GP-related intervals contained 205 genes, and the VI-related intervals contained 150 genes. The interval harboring the A09:47545163 locus, which was jointly detected for both GI and GP, contained 21 genes. Furthermore, based on functional effect annotation of sequence variants, 166 candidate genes carrying nonsynonymous mutations were identified (**Supplementary Table 9**), which were subsequently used as the basis for GO/KEGG enrichment analysis and candidate gene prioritization.

#### 3.7.1 GO and KEGG enrichment analyzes

**Figure 8:**
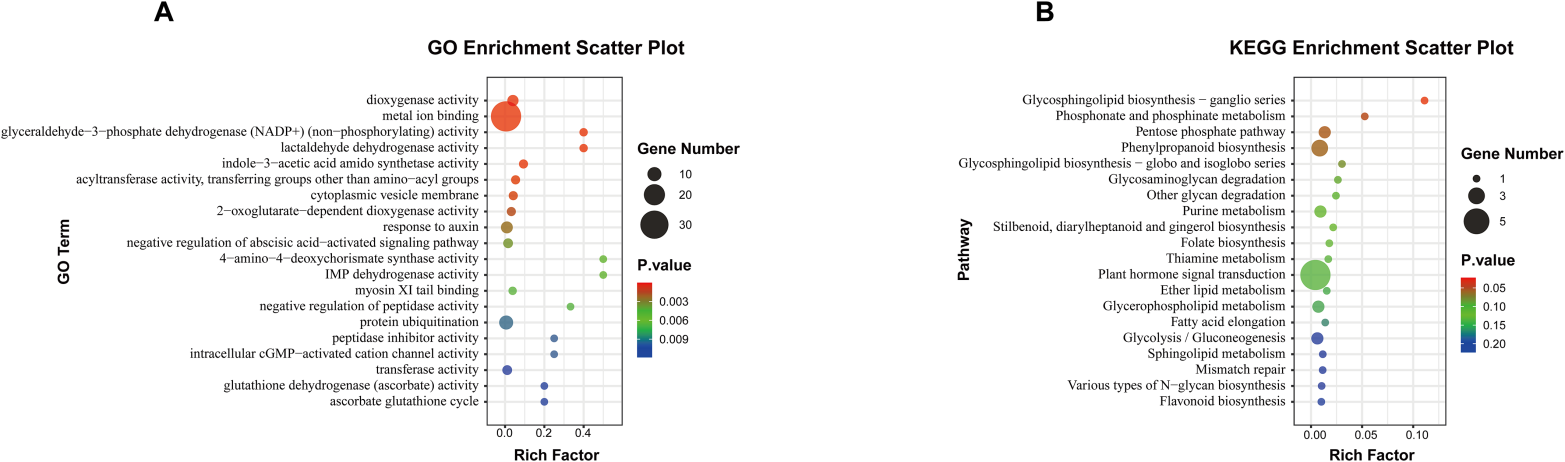
GO and KEGG enrichment analyzes of the 166 non−synonymous mutation genes. **(A)**, GO enrichment analysis. Bubble size represents the number of genes enriched in the corresponding GO term, and color indicates the significance level of enrichment. **(B)**, KEGG enrichment analysis. Bubble size represents the number of genes enriched in the corresponding pathway, and color indicates the significance level of enrichment.

To further elucidate the biological processes and regulatory pathways potentially involving candidate genes underlying the significant association loci, GO and KEGG enrichment analyzes were performed for the 166 genes harboring nonsynonymous mutations (**Figures 8A, B**). A total of 365 GO terms were identified, including 191 Biological Process (BP) terms, 117 Molecular Function (MF) terms, and 57 Cellular Component (CC) terms. In terms of statistical significance, 90 terms reached P< 0.05 and 7 terms reached Q< 0.05, indicating relatively clear enrichment signals for these candidate genes at the GO functional level. KEGG enrichment analysis identified 49 pathways in total, among which several metabolic and signaling pathways closely related to seed vigor could be recognized based on pathway composition and biological function.

Based on the GO/KEGG enrichment results, the intervals harboring significant loci, gene functional annotation, and biological processes associated with seed vigor formation, candidate genes were further prioritized in this study. As a result, 64 candidate genes related to seed vigor were identified, among which *Gh_D04G027200*, *Gh_A10G216900*, *Gh_D05G054900*, *Gh_D05G061300*, *Gh_D05G065300*, *Gh_D13G231700*, *Gh_D13G231900*, *Gh_D13G232500*, *Gh_D05G060800*, and *Gh_D04G026600* were considered top-priority genes for subsequent investigation (**Table 3**). Specifically, *Gh_D04G027200* was associated with auxin response and membrane transport processes, suggesting that it may participate in germination initiation and cellular activation. *Gh_A10G216900* was enriched in terms of ascorbate and glutathione metabolism, indicating a possible role in reactive oxygen species scavenging and maintenance of seed vigor. Both *Gh_D05G054900* and *Gh_D05G061300* were related to the pentose phosphate pathway and glycolysis/gluconeogenesis, suggesting that they may regulate seed vigor by influencing the early germination energy supply and reducing power generation. *Gh_D05G065300* was involved in the negative regulation of ABA signaling and responses to nitric oxide and hydrogen peroxide, implying a potential role in relieving germination inhibition and maintaining redox balance. *Gh_D13G231700* was associated with both ABA signaling regulation and sucrose response, suggesting that it may participate in the coordinated regulation of hormone and sugar signaling during germination initiation. *Gh_D13G231900* was directly related to sucrose metabolism and may function in reserve mobilization and energy supply during the germination process. In the GO enrichment analysis, *Gh_D13G232500* was significantly enriched in the cytoplasmic vesicle membrane (Q< 0.05), suggesting that it may be involved in the vesicular membrane system and intracellular transport processes, thereby affecting post-germination seedling vigor establishment and root and shoot growth. In addition, this gene was associated with the regulation of secondary cell wall biogenesis. *Gh_D05G060800* was related to both lipid metabolism and auxin response, suggesting that it may participate in membrane system repair after seed imbibition and in the regulation of early growth. *Gh_D04G026600* was mainly enriched in auxin response and plant hormone signal transduction, indicating that it may play a regulatory role in germination initiation and radicle elongation. Overall, these 10 prioritized candidate genes were mainly involved in key biological processes, such as hormone signaling regulation, antioxidant defense, energy metabolism, carbohydrate metabolism, and membrane system reconstruction. These functions provide a plausible molecular explanation for the formation of the germination potential, germination index, and vigor index. Therefore, these genes may serve as important targets for subsequent validation and provide valuable clues for identifying key regulatory genes that control seed vigor in cotton.

**Table 3:** Gene function annotation.

Gene name	Gene annotation
*Gh_D04G027200*	Actin 7 family protein, involved in germination and root growth
*Gh_A10G216900*	Dehydroascorbate reductase, involved in AsA–GSH cycle and ROS scavenging
*Gh_D05G054900*	aNADP-dependent glyceraldehyde-3-phosphate dehydrogenase-related protein
*Gh_D05G061300*	GO enrichment analysis revealed significant enrichment of molecular functions related to glyceraldehyde-3-phosphate dehydrogenase, and KEGG annotation assigned it to the pentose phosphate pathway as well as glycolysis/gluconeogenesis pathways, implying a potential role in regulating energy metabolism during early seed germination.
*Gh_D05G065300*	Histidine kinase-related protein, possibly involved in signal transduction
*Gh_D13G231700*	Seven-transmembrane domain protein, possibly involved in membrane signaling
*Gh_D13G231900*	Encodes a protein with putative sucrose-phosphate synthase activity.
*Gh_D13G232500*	Ankyrin and DHHC-CRD domain-containing protein, related to membrane/cell expansion
*Gh_D05G060800*	Encodes an acyltransferase that can modify brassinosteroids (BRs) by acylation and may modulate endogenous BR levels.
*Gh_D04G026600*	Encodes an IAA-amido synthase that conjugates Asp and other amino acids to auxin *in vitro*. Lines carrying insertions in this gene are hypersensitive to auxin.

## 4 Discussion

### 4.1 Importance of seed vigor in agricultural production

Seed vigor refers to the overall potential of seeds to germinate rapidly and uniformly and develop into normal seedlings under a wide range of field conditions. This is specifically reflected in the rate and uniformity of seed germination and seedling growth, speed and uniformity of field emergence and subsequent growth, maintenance of germination capacity after storage and transport, and tolerance to adverse environmental stresses (Chao et al., 2022). With the widespread adoption of mechanized sowing and harvesting in cotton production, the requirements for cotton seed quality have become increasingly stringent. The ability of seeds to germinate rapidly and uniformly in the field is an essential prerequisite for high cotton yield, as well as for mechanized precision sowing and harvesting (Li et al., 2021). Therefore, the level of cotton seed vigor not only affects growth and development throughout the entire growth period and influences field management practices but also has a substantial impact on economic returns. Therefore, investigating the genetic mechanisms underlying seed vigor is of considerable importance. Seed germination vigor is influenced by many factors, including intrinsic genetic factors and external factors such as environmental conditions during germination and growth, seed maturity, mechanical damage during harvesting and processing, and storage conditions (Tan et al., 2025). Because genetic and environmental factors act jointly, many of these influences cannot be effectively selected under conventional breeding strategies (Dai et al., 2022). Furthermore, seed-vigor-related traits are not only controlled by the genotype itself but are also highly sensitive to changes in environmental conditions. Therefore, the use of multi-environment combined phenotypic analysis is necessary in genetic studies. In the present study, 259 Gossypium hirsutum germplasm accessions were phenotyped in seven environments over two years. The results showed that different seed-vigor traits varied markedly in their stability under multi-environment conditions, indicating that both environmental effects and genotype-by-environment interaction effects contributed to the formation of these traits. These findings indicate that association analysis based on multi-environment phenotypic data can not only improve the reliability of phenotypic evaluation but also enhance the stability and interpretability of significant association signals.

### 4.2 GWAS analysis of seed vigor traits in upland cotton

Because seed-vigor-related traits are typically complex quantitative traits whose phenotypic expression is jointly influenced by genotype, environment, and genotype-by-environment interaction, it is necessary to first evaluate the stability and genetic basis of multi-environment phenotypic data before conducting GWAS. Based on the combined phenotypic data from seven environments over two years, the present study found that different traits varied markedly in terms of their genetic stability across environments. Among them, GI, GP, and VI exhibited relatively high genetic explanatory power and good cross-environment consistency. Simultaneously, these three traits also performed prominently in correlation analysis, principal component analysis, and random forest importance evaluation, and were therefore ultimately selected as the key phenotypic traits for subsequent GWAS. With continued development, GWAS has become a powerful and widely used tool for investigating the genetic mechanisms of quantitative traits in crops. Based on linkage disequilibrium within a population, GWAS detects genetic polymorphisms across the whole genome in association panels comprising hundreds of individuals, thereby identifying molecular markers associated with the target phenotypic traits. This approach provides support for marker-assisted selection and target gene identification and offers new technical avenues for crop genetic improvement and breeding practices (Tibbs Cortes et al., 2021; Shenglin et al., 2024). Currently, studies on seed and seedling vigor in Gossypium hirsutum have mainly focused on the genetic mechanisms underlying stress conditions. Gu et al. (2021) constructed a recombinant inbred line population using highly salt-tolerant Gossypium hirsutum cultivars as parental materials and identified a stable salt-tolerance QTL during the seed germination stage, designated qSalt-A04-1, on chromosome A04. Wei et al. (2025) conducted a genome-wide association analysis of seedling traits in 419 Gossypium hirsutum natural accessions under low-phosphorus conditions and ultimately identified six regulatory genes responsive to environmental stress as potential candidate genes for low-phosphorus stress response, located on chromosomes A08, D01, D03, D09, and D12. Aamir Ali Abro et al. (2025) investigated cold tolerance in a population of 302 Gossypium hirsutum accessions and identified two genes associated with seedling cold tolerance on chromosome A11. Shen et al. (2025) performed a GWAS on 383 cotton germplasm accessions for cold tolerance during germination and the seedling stage and identified a novel QTL on chromosome A08. In contrast, relatively few studies have focused on the genetic basis of seed germination and vigor traits under non-stress conditions.

In the present study, GWAS was performed on three seed-vigor-related traits using seeds harvested from 259 Gossypium hirsutum germplasm accessions grown in seven environments over two years. A total of 15 SNPs were repeatedly detected in at least two environments and were identified on seven different chromosomes of Gossypium hirsutum (**Table 1**). Compared with previous studies, the five loci identified on chromosome A10 in this study (A10:109096218, A10:109120366, A10:109120427, A10:109124473, and A10:109124660) were located in close genomic proximity to locus A10:112752002 identified by Li et al. (2023) in a natural population of 355 Gossypium hirsutum accessions. On chromosome A08, the SNP locus A08:56855259 identified in this study was also located near locus A08:53492125 detected by Si et al. (2022) in 419 core Gossypium hirsutum germplasm accessions. These results suggest that chromosomes A10 and A08 may harbor key genetic factors that conservatively regulate seed germination vigor across different genetic backgrounds. Notably, both Li et al. (2023) and Si et al. (2022)previously identified significant loci associated with seed vigor on chromosome A09, and the significant locus A09:47545163, which was repeatedly detected in the present study for both germination index and germination, was also located on this chromosome, further supporting the important role of chromosome A09 in the regulation of seed vigor. In addition to these findings near previously reported loci, this study also identified a series of novel SNPs showing stable associations across different environments that may be involved in seed vigor regulation, including D05:4875896 on chromosome D05, D13:5100227 and D13:60741776 on chromosome D13, A02:91069897 on chromosome A02, and D04:3372587, D04:3373713, and D04:3374750 on chromosome D04.

### 4.3 Functions and prediction of candidate genes

A large body of evidence has shown that seed germination in plants is regulated by numerous endogenous and exogenous factors, among which hormone signaling, redox homeostasis, reserve mobilization, and membrane system reconstruction are key processes that affect germination initiation and vigor maintenance. Hormones, particularly abscisic acid (ABA) and gibberellin (GA), play central roles in seed germination. The coordinated balance between these two hormones is crucial for germination, with ABA maintaining seed dormancy and inhibiting germination, whereas GA antagonizes ABA and promotes germination (Finkelstein et al., 2008; Abley et al., 2021; Chen et al., 2021; Liao et al., 2023). At the same time, auxin also extensively interacts with ABA and participates in the regulation of radicle protrusion, root elongation, and post-germination seedling establishment (Liu et al., 2013). Based on the candidate gene screening results of the present study, 10 genes, namely *Gh_D04G027200, Gh_A10G216900, Gh_D05G054900, Gh_D05G061300, Gh_D05G065300, Gh_D13G231700, Gh_D13G231900, Gh_D13G232500, Gh_D05G060800*, and *Gh_D04G026600*, were prioritized as candidate genes potentially associated with cotton seed vigor. Among these, *Gh_D04G027200* encodes actin 7 and belongs to the actin gene family. Previous studies have shown that ACT7 in Arabidopsis thaliana plays an important role in seed germination and root growth, and its mutants exhibit impaired germination and defective root development. ACT7 is also involved in auxin-mediated regulation of root meristem development and cell elongation (Gilliland et al., 2003; Numata et al., 2022). Combined with the annotation results of this study linking this gene to auxin response, protein transport, and cell wall-related processes, *Gh_D04G027200* is likely involved in cotton seed vigor formation through the regulation of cytoskeletal dynamics and post-germination growth processes. *Gh_A10G216900* encodes dehydroascorbate reductase 2 (DHAR2), a glutathione-dependent dehydroascorbate reductase. Previous studies have demonstrated that the ascorbate-glutathione cycle is an important antioxidant system during early seed germination for scavenging reactive oxygen species (ROS), maintaining mitochondrial function, and delaying vigor deterioration. In cotton, ascorbic acid priming has been shown to improve low-temperature germination by enhancing membrane stability and antioxidant cycling (Han et al., 2025). Therefore, *Gh_A10G216900* is likely to affect the maintenance of cotton seed vigor and germination ability under stress by participating in ROS scavenging, alleviating membrane lipid peroxidation, and maintaining redox homeostasis. Both *Gh_D05G054900* and *Gh_D05G061300* were associated with glyceraldehyde-3-phosphate dehydrogenase (NADP+) (non-phosphorylating) activity in the GO analysis and were jointly enriched in the pentose phosphate pathway and glycolysis/gluconeogenesis pathways in the KEGG analysis. Previous studies have shown that during early germination, sugar metabolic reprogramming, the transition between fermentation and respiratory metabolism, and the rapid mobilization of storage substances are important determinants of germination efficiency and seedling establishment (Bharadwaj et al., 2021; El-Maarouf-Bouteau, 2022). Therefore, *Gh_D05G054900* and *Gh_D05G061300* are likely to participate in cotton seed vigor formation by regulating early germination energy supply, carbon allocation, and reducing power generation. *Gh_D05G065300* was mainly associated with the negative regulation of the abscisic acid-activated signaling pathway, cellular response to hydrogen peroxide, and cellular response to nitric oxide. Previous studies have shown that ABA is a key hormone that inhibits seed germination, whereas H_2_O_2_ and NO can promote dormancy release and germination by regulating ABA catabolism, GA biosynthesis, and the signaling balance. Moreover, H_2_O_2_/Ca²^+^ signaling crosstalk can promote seed germination under ABA stress (Ali et al., 2022; Cheng et al., 2022). Therefore, *Gh_D05G065300* is likely positioned at a key regulatory node involving “release from ABA-mediated inhibition and ROS/NO signaling balance.” The significant loci identified on chromosome D13 (D13:60741776 and D13:5100227) were located on the same chromosome as significant loci previously reported by Si et al. (2022). *Gh_D13G231700* and *Gh_D13G231900* mainly regulate sugar signaling and sucrose metabolism. Specifically, *Gh_D13G231700* was enriched in the negative regulation of the abscisic acid-activated signaling pathway and response to sucrose, suggesting that it may participate in the coordinated regulation of ABA and sugar signaling. In contrast, *Gh_D13G231900* was significantly associated with sucrose-phosphate synthase activity, sucrose synthase activity, sucrose biosynthetic processes, and starch and sucrose metabolism. Previous studies have shown that sucrose is not only an important carbon source for post-germination seedling establishment but also a key signaling molecule regulating germination and early growth. SUS, SPS, and sucrose transport systems jointly participate in carbon allocation, cell wall synthesis, and early seedling growth (Maloney et al., 2015; Stein and Granot, 2019). Therefore, *Gh_D13G231700* is likely to influence germination initiation by mediating ABA/sucrose signaling crosstalk, whereas *Gh_D13G231900* may mainly participate in seed vigor formation by regulating sucrose synthesis, reserve mobilization, and energy supply during germination. *Gh_D13G232500* represents a functional direction related to membrane transport and cell wall biogenesis. In the GO enrichment analysis, this gene was mainly associated with the cytoplasmic vesicle membrane and regulation of secondary cell wall biogenesis. Combined with its annotation as encoding a protein containing ankyrin and DHHC-CRD domains, it is likely involved in membrane localization, vesicle transport, and cell wall component deposition. Previous studies have shown that vesicle transport and cell wall formation are closely related to radicle elongation, cell expansion, and early seedling establishment after germination (Hemsley et al., 2005; Luo et al., 2022). Accordingly, *Gh_D13G232500* may participate in post-germination seedling vigor formation in cotton by regulating membrane transport and cell wall biogenesis. *Gh_D04G026600* was mainly enriched in response to auxin and plant hormone signal transduction, suggesting a role that is more specifically related to auxin-mediated post-germination growth regulation. Previous studies have shown that auxin is not only involved in the regulation of seed dormancy and germination, but also affects post-germination seedling establishment by modulating ABA signaling, root meristem development, and cell elongation. Oxidative stress and water-deficit signaling are also important factors influencing seed vigor and germination ability under stress (Liu et al., 2013; Cheng et al., 2022). Accordingly, *Gh_D13G236400* may coordinate auxin, redox, and water stress responses under adverse conditions, whereas *Gh_D04G026600* may participate more directly in germination initiation and radicle elongation in cotton seeds. *Gh_D05G060800* was mainly enriched in GO terms such as acyltransferase activity, transferring groups other than amino-acyl groups and long-chain fatty acid-CoA ligase activity, and was associated with lipid metabolism-related pathways. Previous studies have shown that marked membrane phospholipid remodeling and repair occur during seed imbibition. In cotton, membrane lipid unsaturation and glycerophospholipid composition directly affect membrane stability and germination capacity during low-temperature imbibition. Long-chain acyl-CoA synthetases and lipid metabolism-related enzymes participate in fatty acid activation, β-oxidation, and the metabolic conversion of the auxin precursor IBA to IAA, thereby influencing root elongation and early growth (Lin et al., 2019; Jawahir and Zolman, 2021; Dhaliwal et al., 2024; Dhaliwal et al., 2025). Therefore, *Gh_*D05G060800 is likely to affect vigor maintenance during cotton seed imbibition and stress germination by participating in membrane lipid metabolism, membrane system repair and lipid-hormone interactions.

In this study, public transcriptomic expression data for TM-1 from the CottonMD database were used as exploratory supporting evidence for inferring candidate gene function (**Supplementary Table 10**). The public expression profiles showed that several prioritized candidate genes, including *Gh_*D04G027200, *Gh_*D05G060800, *Gh_*D05G065300, Gh_D13G231700, Gh_A10G216900, and *Gh_*D13G231900, exhibited measurable expression responses in roots, seedling tissues, or under stress treatments. These expression patterns were broadly consistent with their functional annotations related to hormone regulation, antioxidant defense, energy metabolism, and membrane system remodeling, thereby providing a useful reference for subsequent qRT-PCR assays and genetic functional validation.

Overall, these 10 candidate genes mainly point to functional processes including cytoskeleton and auxin regulation, ABA/ROS signaling balance, the ascorbate-glutathione cycle, sugar metabolism and reserve mobilization, as well as membrane lipid remodeling and maintenance of membrane system stability. These processes correspond to key stages in seed vigor formation, including germination initiation, dormancy release, antioxidant protection, energy supply, and early seedling establishment. Combined with the expression pattern analysis based on transcriptomic data from different TM-1 tissues/organs and under low-temperature, high-temperature, PEG, and NaCl treatments from the CottonMD database, these 10 genes are likely to jointly participate in cotton seed vigor formation and stress responses and, therefore, can be regarded as priority candidate genes for subsequent functional validation.

## 5 Conclusions

Through multi-environment phenotypic evaluation and genome-wide association analysis of 259 Gossypium hirsutum germplasm accessions, this study systematically dissected the genetic basis of cotton seed germination vigor. A total of 15 SNPs showing stable associations across different environments were identified, and 10 genes, namely *Gh_*D04G027200, *Gh_*A10G216900, *Gh_*D05G054900, *Gh_D05G061300*, *Gh_*D05G065300, *Gh_*D13G231700, *Gh_*D13G231900, *Gh_D13G232500*, *Gh_D05G060800*, and *Gh_D04G026600*, were highlighted as key candidate genes potentially involved in the regulation of cotton seed vigor. Comparison with previous studies indicated that chromosomes A10, A08, A09, and D13 harbor conserved genetic regions regulating seed germination and vigor, suggesting that these regions may contain important genetic factors that function across different genetic backgrounds. Overall, this study provides new theoretical evidence for elucidating the genetic mechanisms underlying cotton seed germination vigor and offers important gene resources and references for subsequent functional gene mining and molecular breeding improvement in cotton.

## Data Availability

The original contributions presented in the study are included in the article/**Supplementary Material**. Further inquiries can be directed to the corresponding author.

## Supplementary material

Supplementary Table S1 Collection of cotton varieties and their geographical origins.

Supplementary Table S2 Descriptive statistics of germination-related traits across seven environments.

Supplementary Table S3 Variance analysis for SV-related traits in the natural population.

Supplementary Table S4 Significant SNP Loci for the Germination Index (GI).

Supplementary Table S5 Significant SNP Loci for the Germination Potential (GP).

Supplementary Table S6 Significant SNP Loci for the Vigor Index (VI).

Supplementary Table S7 449 genes within eight merged intervals.

Supplementary Figure S8 (A-O) Violin plots illustrating genotype-phenotype analysis of 15 significant SNP loci for three traits.

Supplementary Figure S9 Functional annotation of 166 non-synonymous mutations.

Supplementary Figure S10 Spatiotemporal expression profiles of 10 key candidate genes based on TM-1 (CRI) RNA‑seq data.

## References

[B1] Abley K. Formosa-Jordan P. Tavares H. Chan E. Y. Afsharinafar M. Leyser O. . (2021). An ABA-GA bistable switch can account for natural variation in the variability of Arabidopsis seed germination time. Elife 10, e59485. doi: 10.7554/eLife.59485. PMID: 34059197 PMC8169117

[B2] Abro A. A. Abbas M. Liu Q. Jie Z. Xu Y. Hou Y. . (2025). Genetic insights into cold tolerance in cotton: GWAS identified GhPRL gene responsible for cold tolerance in cotton at seedling stage. Ind. Crop Prod. 237, 122164. doi: 10.1016/j.indcrop.2025.122164. PMID: 38826717

[B3] Ali F. Qanmber G. Li F. Wang Z . (2022). Updated role of ABA in seed maturation, dormancy, and germination. J. Adv. Res. 35, 199–214. doi: 10.1016/j.jare.2021.03.011. PMID: 35003801 PMC8721241

[B4] Bharadwaj R. Noceda C. Mohanapriya G. Kumar S. R. Thiers K. L. L. Costa J. H. . (2021). Adaptive reprogramming during early seed germination requires temporarily enhanced fermentation-a critical role for alternative oxidase regulation that concerns also microbiota effectiveness. Front. Plant Sci. 12, 686274. doi: 10.3389/fpls.2021.686274. PMID: 34659277 PMC8518632

[B5] Chao L. Pan Z. Wang J. Wu Y. Shui G. Aini N. . (2022). Genetic mapping and analysis of a compact plant architecture and precocious mutant in upland cotton. Plants (Basel Switzerland) 11, 1483. doi: 10.3390/plants11111483. PMID: 35684255 PMC9182648

[B6] Chen L. Lu B. Liu L. Duan W. Jiang D. Li J. . (2021). Melatonin promotes seed germination under salt stress by regulating ABA and GA(3) in cotton (Gossypium hirsutum L.). Plant Physiol. Biochem: PPB 162, 506–516. doi: 10.1016/j.plaphy.2021.03.029. PMID: 33773227

[B7] Chen Z. J. Scheffler B. E. Dennis E. Triplett B. A. Zhang T. Guo W. . (2007). Toward sequencing cotton (Gossypium) genomes. Plant Physiol. 145, 1303–1310. doi: 10.1104/pp.107.107672. PMID: 18056866 PMC2151711

[B8] Cheng M. Guo Y. Liu Q. Nan S. Xue Y. Wei C. . (2022). H(2)O(2) and Ca(2+) signaling crosstalk counteracts ABA to induce seed germination. Antioxidants (Basel Switzerland) 11(8), 1594. doi: 10.3390/antiox11081594. PMID: 36009313 PMC9404710

[B9] Dai L. Lu X. Shen L. Guo L. Zhang G. Gao Z. . (2022). Genome-wide association study reveals novel QTLs and candidate genes for seed vigor in rice. Front. Plant Sci. 13, 1005203. doi: 10.3389/fpls.2022.1005203. PMID: 36388599 PMC9645239

[B10] Deng. Y. H. (2022). Mining and functional identification of drought-resistant genes in upland cotton (Doctoral dissertation) Xinjiang Agricultural University. doi: 10.27431/d.cnki.gxnyu.2022.000097

[B11] Dhaliwal L. K. Osti B. Angeles-Shim R. B . (2025). Phosphatidic acid accumulation in response to extended cold water imbibition disrupts membrane structure that inhibits germination of cotton (Gossypium hirsutum L.) seeds. Curr. Plant Biol. 42, 100491. doi: 10.1016/j.cpb.2025.100491. PMID: 38826717

[B12] Dhaliwal L. K. Shim J. Auld D. Angeles-Shim R. B . (2024). Fatty acid unsaturation improves germination of upland cotton (Gossypium hirsutum) under cold stress. Front. Plant Sci. 15, 1286908. doi: 10.3389/fpls.2024.1286908. PMID: 38379948 PMC10877374

[B13] Dong C. Wang J. Yu Y. Ju L. Zhou X. Ma X. . (2018). Identifying functional genes influencing Gossypium hirsutum fiber quality. Front. Plant Sci. 9, 1968. doi: 10.3389/fpls.2018.01968. PMID: 30687363 PMC6334163

[B14] El-Maarouf-Bouteau H. (2022). The seed and the metabolism regulation. Biology 11(2), 168. doi: 10.3390/biology11020168. PMID: 35205035 PMC8869448

[B15] Finkelstein R. Reeves W. Ariizumi T. Steber C . (2008). Molecular aspects of seed dormancy. Annu. Rev. Plant Biol. 59, 387–415. doi: 10.1146/annurev.arplant.59.032607.092740. PMID: 18257711

[B16] Gilliland L. U. Pawloski L. C. Kandasamy M. K. Meagher R. B . (2003). Arabidopsis actin gene ACT7 plays an essential role in germination and root growth. Plant Journ: For. Cell. Mol. Biol. 33, 319–328. doi: 10.1046/j.1365-313x.2003.01626.x. PMID: 12535345

[B17] Gong D. He F. Liu J. Zhang C. Wang Y. Tian S. . (2022). Understanding of hormonal regulation in rice seed germination. Life. (Basel Switzerland) 12, 1021. doi: 10.3390/life12071021. PMID: 35888110 PMC9324290

[B18] Gu Q. Ke H. Liu C. Lv X. Sun Z. Liu Z. . (2021). A stable QTL qSalt-A04–1 contributes to salt tolerance in the cotton seed germination stage. Tag. Theor. Appl. Genet. Theoretische Und Angewandte Genetik 134, 2399–2410. doi: 10.1007/s00122-021-03831-0. PMID: 33928409

[B19] Han P. Ma H. Lu L. Zhu J. Nie X. Xu J. . (2025). Ascorbic acid priming boosts cotton seed chilling tolerance via membrane stability and antioxidant cycles. Plants (Basel Switzerland) 14(20), 3122. doi: 10.3390/plants14203122. PMID: 41157681 PMC12567305

[B20] Hemsley P. A. Kemp A. C. Grierson C. S (2005). The TIP GROWTH DEFECTIVE1 S-acyl transferase regulates plant cell growth in Arabidopsis. Plant Cell 17, 2554–2563. doi: 10.1105/tpc.105.031237. PMID: 16100337 PMC1197434

[B21] Jawahir V. Zolman B. K. (2021). Long chain acyl CoA synthetase 4 catalyzes the first step in peroxisomal indole-3-butyric acid to IAA conversion. Plant Physiol. 185, 120–136. doi: 10.1093/plphys/kiaa002. PMID: 33631795 PMC8133310

[B22] Li L. Hu Y. Wang Y. Zhao S. You Y. Liu R. . (2023). Identification of novel candidate loci and genes for seed vigor-related traits in upland cotton (Gossypium hirsutum L.) via GWAS. Front. Plant Sci. 14, 1254365. doi: 10.3389/fpls.2023.1254365. PMID: 37719213 PMC10503134

[B23] Li X. Kong X. Zhou J. Luo Z. Lu H. Li W. . (2021). Seeding depth and seeding rate regulate apical hook formation by inducing GhHLS1 expression via ethylene during cotton emergence. Plant Physiol. Biochem: PPB 164, 92–100. doi: 10.1016/j.plaphy.2021.04.030. PMID: 33975148

[B24] Li Y. Liang Y. Liu M. Zhang Q. Wang Z. Fan J. . (2022). Genome-wide association studies provide insights into the genetic architecture of seed germination traits in maize. Front. Plant Sci. 13, 930438. doi: 10.3389/fpls.2022.930438. PMID: 35755688 PMC9226777

[B25] Liao Z. Zhang Y. Yu Q. Fang W. Chen M. Li T. . (2023). Coordination of growth and drought responses by GA-ABA signaling in rice. New Phytol. 240, 1149–1161. doi: 10.1111/nph.19209. PMID: 37602953

[B26] Lin Y. X. Xin X. Yin G. K. He J. J. Zhou Y. C. Chen J. Y. . (2019). Membrane phospholipids remodeling upon imbibition in Brassica napus L. seeds. Biochem. Bioph Res. Co 515, 289–295. doi: 10.1016/j.bbrc.2019.05.100. PMID: 31146920

[B27] Liu L. Ma Y. Zhao H. Guo L. Guo Y. Liu C. M. . (2024). Genome-wide association studies identified OsTMF as a gene regulating rice seed germination under salt stress. Front. Plant Sci. 15, 1384246. doi: 10.3389/fpls.2024.1384246. PMID: 38601316 PMC11004275

[B28] Liu X. Zhang H. Zhao Y. Feng Z. Li Q. Yang H. Q. . (2013). Auxin controls seed dormancy through stimulation of abscisic acid signaling by inducing ARF-mediated ABI3 activation in Arabidopsis. P Natl. Acad. Sci. U.S.A. 110, 15485–15490. doi: 10.1073/pnas.1304651110. PMID: 23986496 PMC3780901

[B29] Luo C. Shi Y. Xiang Y. (2022). SNAREs regulate vesicle trafficking during root growth and development. Front. Plant Sci. 13, 853251. doi: 10.3389/fpls.2022.853251. PMID: 35360325 PMC8964185

[B30] Ma L. Wang C. Hu Y. Dai W. Liang Z. Zou C. . (2022). GWAS and transcriptome analysis reveal MADS26 involved in seed germination ability in maize. Tag. Theor. Appl. Genet. Theoretische Und Angewandte Genetik 135, 1717–1730. doi: 10.1007/s00122-022-04065-4. PMID: 35247071

[B31] Maloney V. J. Park J. Y. Unda F. Mansfield S. D . (2015). Sucrose phosphate synthase and sucrose phosphate phosphatase interact in planta and promote plant growth and biomass accumulation. J. Exp. Bot. 66, 4383–4394. doi: 10.1093/jxb/erv101. PMID: 25873678 PMC4493782

[B32] Mohammed J. Thyssen G. N. Hinze L. Zhang J. Zeng L. Fang D. D. . (2024). A GWAS identified loci and candidate genes associated with fiber quality traits in a new cotton MAGIC population. Tag. Theor. Appl. Genet. Theoretische Und Angewandte Genetik 138, 10. doi: 10.1007/s00122-024-04800-z. PMID: 39714714

[B33] Numata T. Sugita K. Ahamed Rahman A. Rahman A . (2022). Actin isovariant ACT7 controls root meristem development in Arabidopsis through modulating auxin and ethylene responses. J. Exp. Bot. 73, 6255–6271. doi: 10.1093/jxb/erac280. PMID: 35749807

[B34] Purcell S. Neale B. Todd-Brown K. Thomas L. Ferreira M. A. Bender D. . (2007). PLINK: a tool set for whole-genome association and population-based linkage analyses. Am. J. Hum. Genet. 81, 559–575. doi: 10.1086/519795. PMID: 17701901 PMC1950838

[B35] Qian J. F. Song Y. L. Yuan R. L. Feng L. (2020). China′s cotton industry safety issues and development strategies under open conditions. J. Agric. Resour. Regionalization China 41, 140–145. Available online at: https://link.cnki.net/urlid/11.3513.S.20200701.1101.004

[B36] Shen Q. Zhang S. Ge C. Liu S. Chen J. Liu R. . (2023). Genome-wide association study identifies GhSAL1 affects cold tolerance at the seedling emergence stage in upland cotton (Gossypium hirsutum L.). Tag. Theor. Appl. Genet. Theoretische Und Angewandte Genetik 136, 27. doi: 10.1007/s00122-023-04317-x. PMID: 36810826

[B37] Shen W. Li S. Zhou B. Li J. Dong Y. Liu R. . (2025). Genome-wide association analysis revealed that GhD6PKL2 regulates cold tolerance at seed germination and seedling emergence in cotton. Environ. Exp. Bot. 241, 106292. doi: 10.1016/j.envexpbot.2025.106292. PMID: 38826717

[B38] Shenglin R. Caiwen W. Yanfen J. Jiayong L. (2024). Research progress on genomic association studies in crops. Mol. Plant Breed. 22, 3594–3602. Available online at: https://link.cnki.net/doi/10.13271/j.mpb.022.003594

[B39] Shi Y. Gao L. Wu Z. Zhang X. Wang M. Zhang C. . (2017). Genome-wide association study of salt tolerance at the seed germination stage in rice. BMC Plant Biol. 17, 92. doi: 10.1186/s12870-017-1044-0. PMID: 28558653 PMC5450148

[B40] Si A. Sun Z. Li Z. Chen B. Gu Q. Zhang Y. . (2022). A genome wide association study revealed key single nucleotide polymorphisms/genes associated with seed germination in Gossypium hirsutum L. Front. Plant Sci. 13, 844946. doi: 10.3389/fpls.2022.844946. PMID: 35371175 PMC8967292

[B41] Song X. Zhu G. Hou S. Ren Y. Amjid M. W. Li W. . (2021). Genome-wide association analysis reveals loci and candidate genes involved in fiber quality traits under multiple field environments in cotton (Gossypium hirsutum). Front. Plant Sci. 12, 695503. doi: 10.3389/fpls.2021.695503. PMID: 34421946 PMC8374309

[B42] Stein O. Granot D. (2019). An overview of sucrose synthases in plants. Front. Plant Sci. 10, 95. doi: 10.3389/fpls.2019.00095. PMID: 30800137 PMC6375876

[B43] Sun Z. Li H. Zhang Y. Li Z. Ke H. Wu L. . (2018). Identification of SNPs and candidate genes associated with salt tolerance at the seedling stage in cotton (Gossypium hirsutum L.). Front. Plant Sci. 9, 1011. doi: 10.3389/fpls.2018.01011. PMID: 30050555 PMC6050395

[B44] Tan S. Cao J. Li S. Li Z . (2025). Unraveling the mechanistic basis for control of seed longevity. Plants (Basel Switzerland) 14, 805. doi: 10.3390/plants14050805. PMID: 40094799 PMC11902243

[B45] Thyssen G. N. Jenkins J. N. McCarty J. C. Zeng L. Campbell B. T. Delhom C. D. . (2019). Whole genome sequencing of a MAGIC population identified genomic loci and candidate genes for major fiber quality traits in upland cotton (Gossypium hirsutum L.). Tag. Theor. Appl. Genet. Theoretische Und Angewandte Genetik 132, 989–999. doi: 10.1007/s00122-018-3254-8. PMID: 30506522

[B46] Tibbs Cortes L. Zhang Z. Yu J (2021). Status and prospects of genome-wide association studies in plants. Plant Genome 14, e20077. doi: 10.1002/tpg2.20077. PMID: 33442955 PMC12806871

[B47] Wang L. Yang Y. Qin J. Ma Q. Qiao K. Fan S. . (2025). Regulation of seed germination: ROS, epigenetic, and hormonal aspects. J. Adv. Res. 71, 107–125. doi: 10.1016/j.jare.2024.06.001. PMID: 38838783 PMC12126707

[B48] Wang Y. Sun X. Peng J. Li F. Ali F. Wang Z. . (2025). Integrative GWAS and transcriptomics reveal GhAMT2 as a key regulator of cotton resistance to Verticillium wilt. Front. Plant Sci. 16, 1563466. doi: 10.3389/fpls.2025.1563466. PMID: 40353226 PMC12062179

[B49] Wei X. Yao S. Di J. Guan J. Wang A. Yang J. . (2025). Single- and multi-locus GWAS unravels novel genomic regions related to low-phosphate stress in cotton seedlings. Plants (Basel Switzerland) 14, 1803. doi: 10.3390/plants14121803. PMID: 40573790 PMC12196663

[B50] Yang Z. Wang J. Huang Y. Wang S. Wei L. Liu D. . (2023). CottonMD: a multi-omics database for cotton biological study. Nucleic Acids Res. 51, D1446–D1456. doi: 10.1093/nar/gkac863. PMID: 36215030 PMC9825545

[B51] Yuan T. Y. Wang J. H. Zhang S. Q. Du J. Li C. Yan J. . (2025). Screening of high-yield and high-quality wheat varieties based on principal component analysis. Seed 44, 175–182. doi: 10.16590/j.cnki.1001-4705.2025.10.175

[B52] Zhang Y. Luo J. Feng S. Ke X. Jia H. Zhu Q. . (2024). Prediction of the fluoride contents of different crop species via the random forest algorithm. Environ. Geochem. Hlth 46, 418. doi: 10.1007/s10653-024-02206-w. PMID: 39249634

[B53] Zhao H. Zhang Y. Zheng Y. (2022). Integration of ABA, GA, and light signaling in seed germination through the regulation of ABI5. Front. Plant Sci. 13, 1000803. doi: 10.3389/fpls.2022.1000803. PMID: 36092418 PMC9449724

