# Supplementary material for: Genome-wide association study identifies key loci and candidate genes for seed vigor in upland cotton (Gossypium hirsutum L.)
Source: Front Plant Sci. 2026 Jun 1;17:1804577. doi: 10.3389/fpls.2026.1804577 (PMC13265474; doi:10.3389/fpls.2026.1804577)
Supplement: Supplementary Table S1 — Collection of cotton varieties and their geographical origins. [file SupplementaryFile1.zip › Figure. S8.docx]

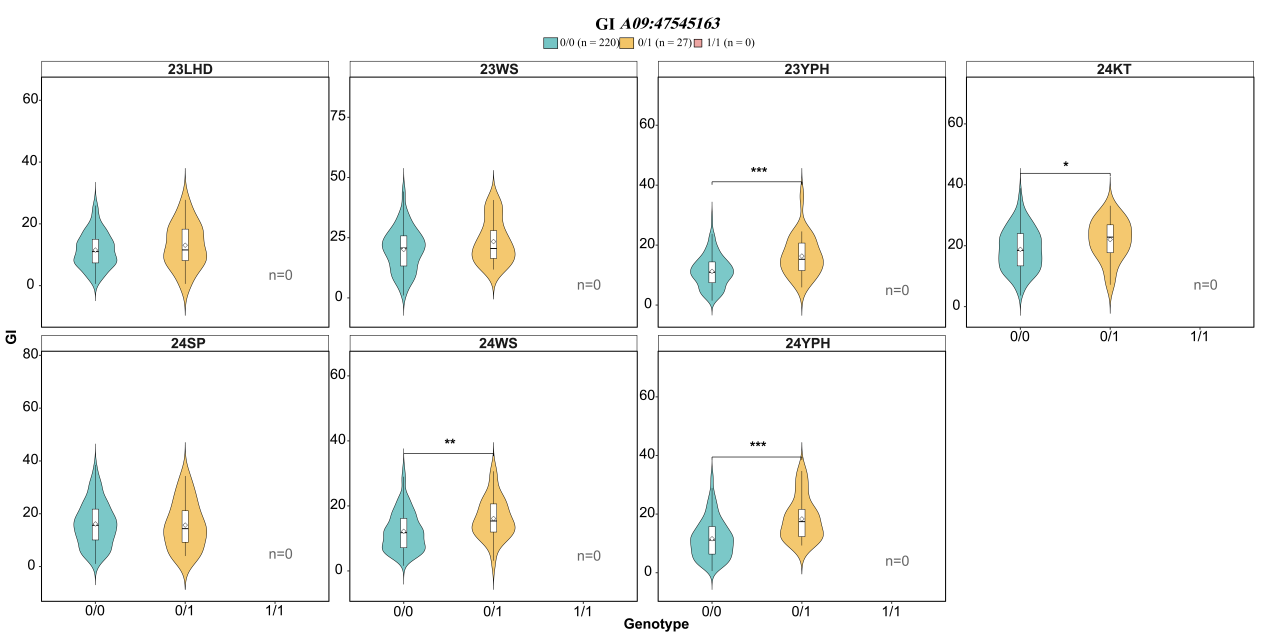

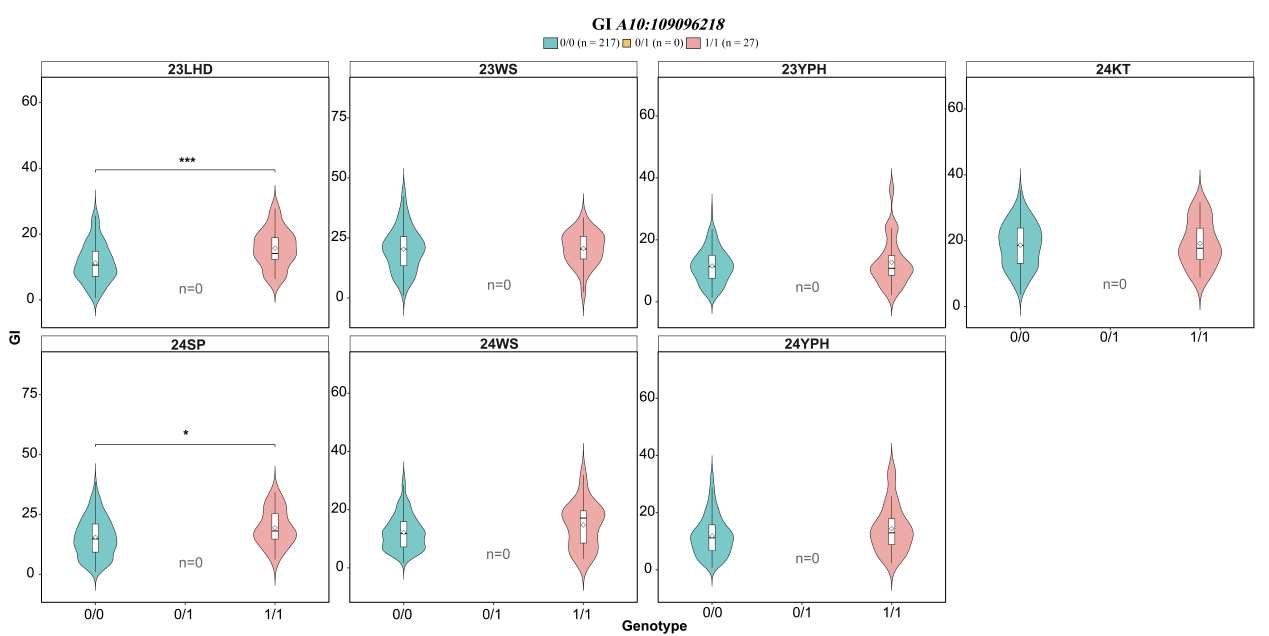

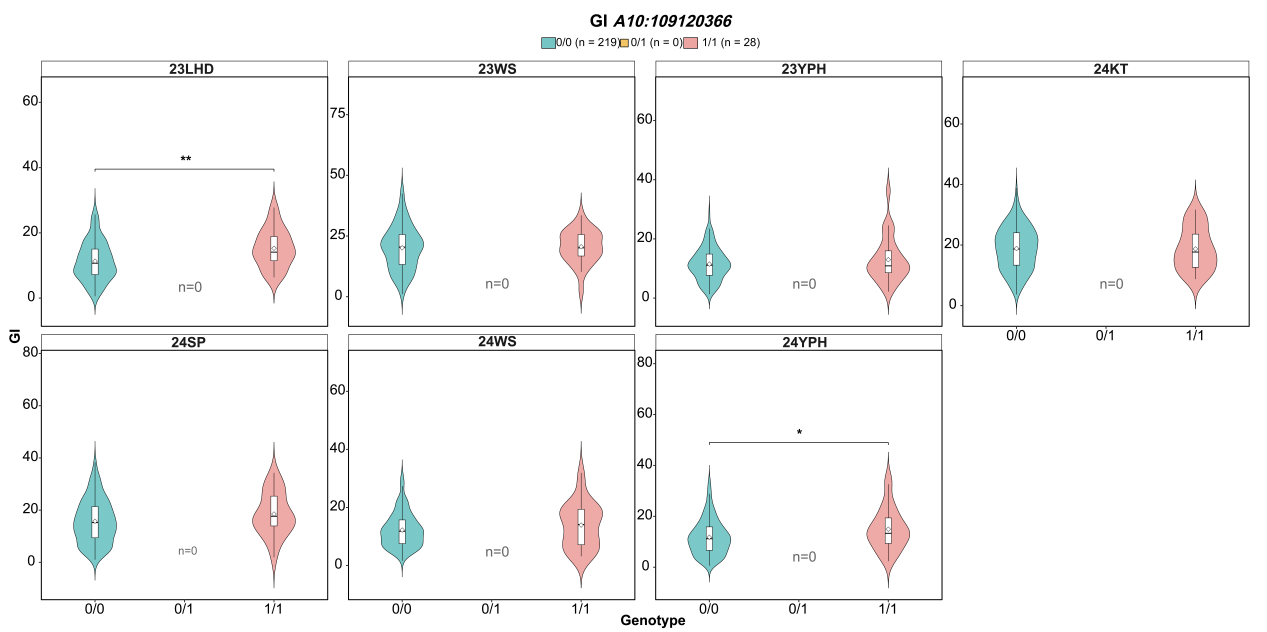

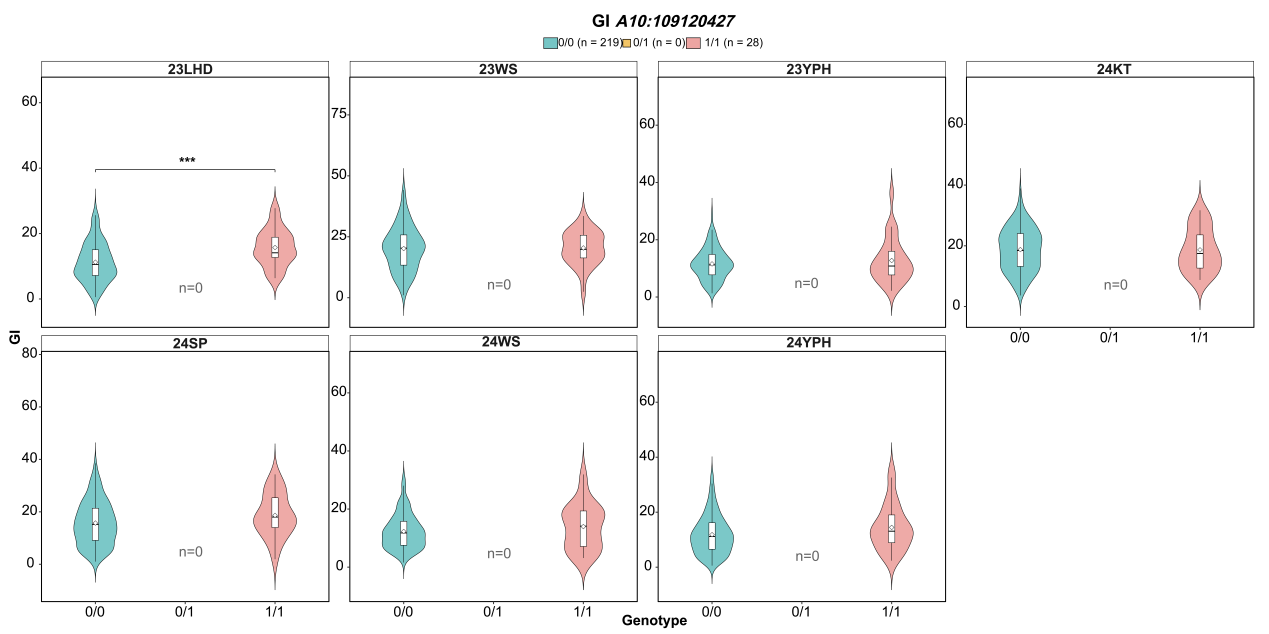

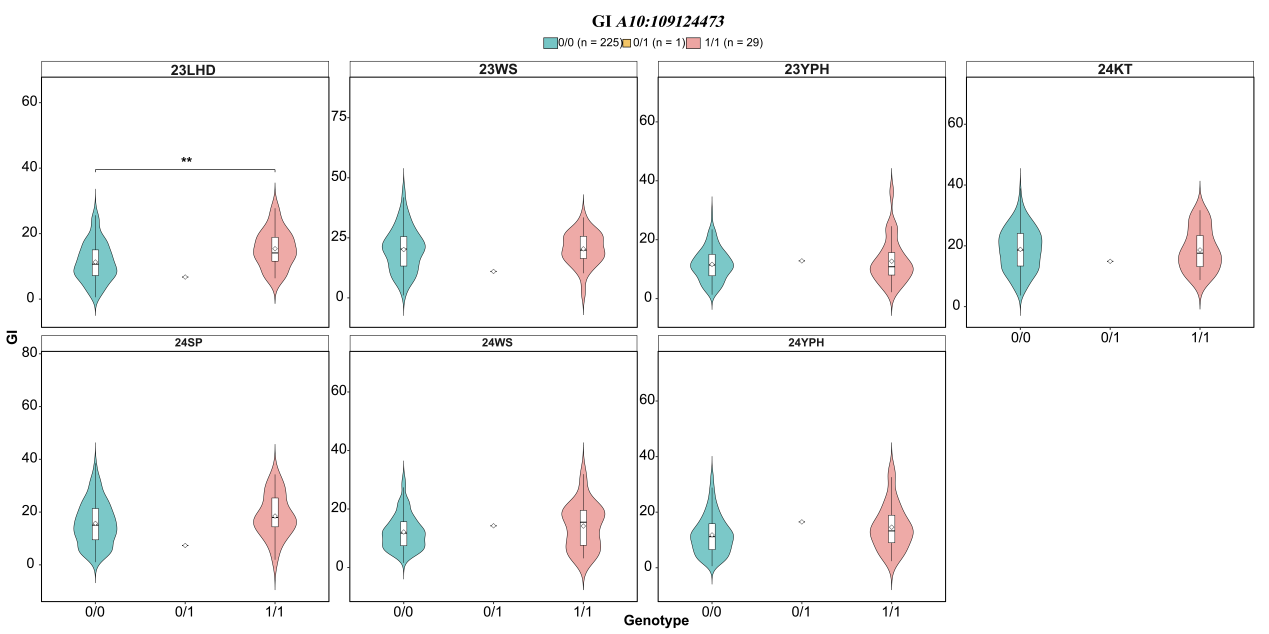

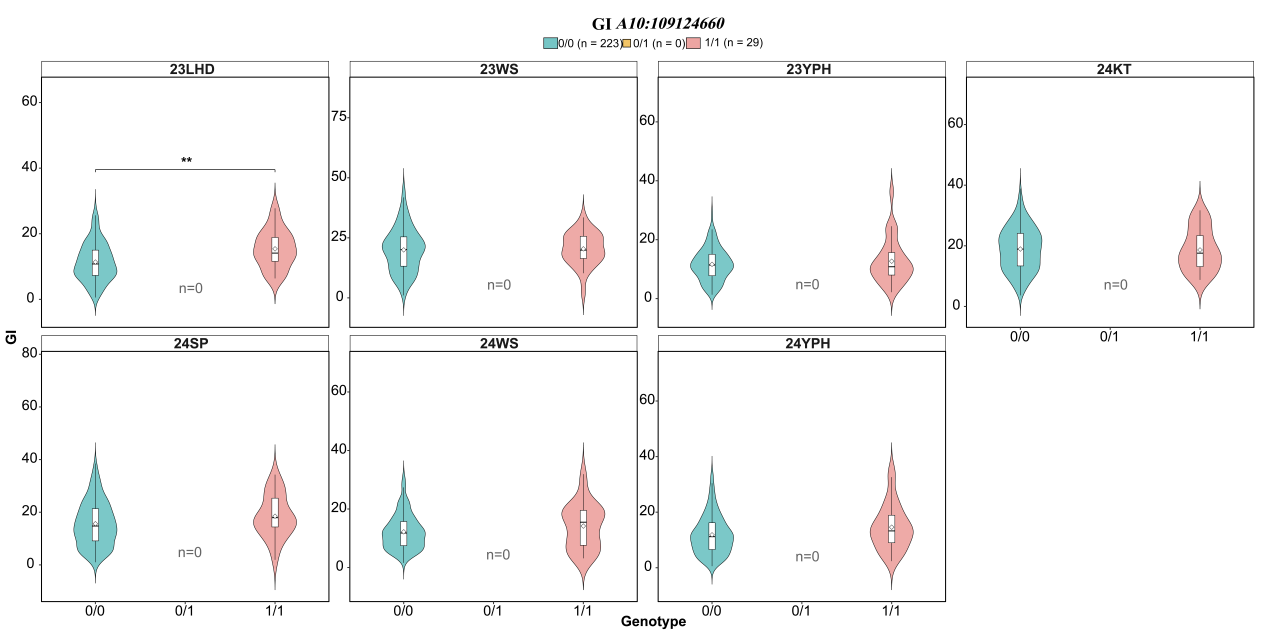

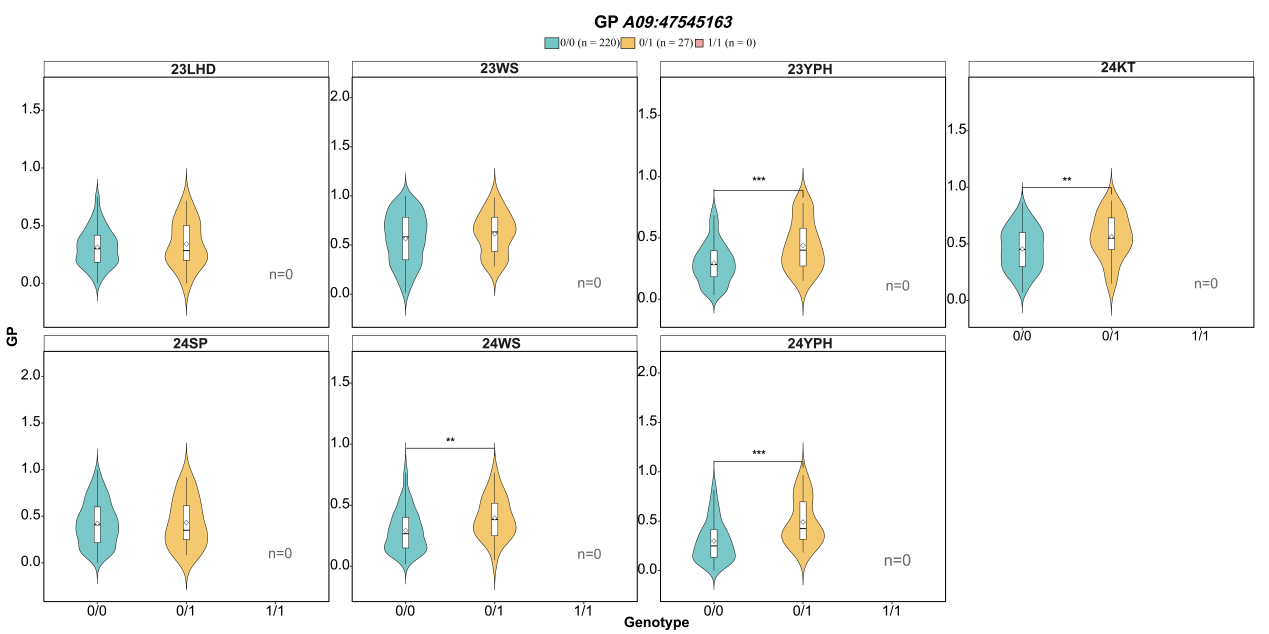

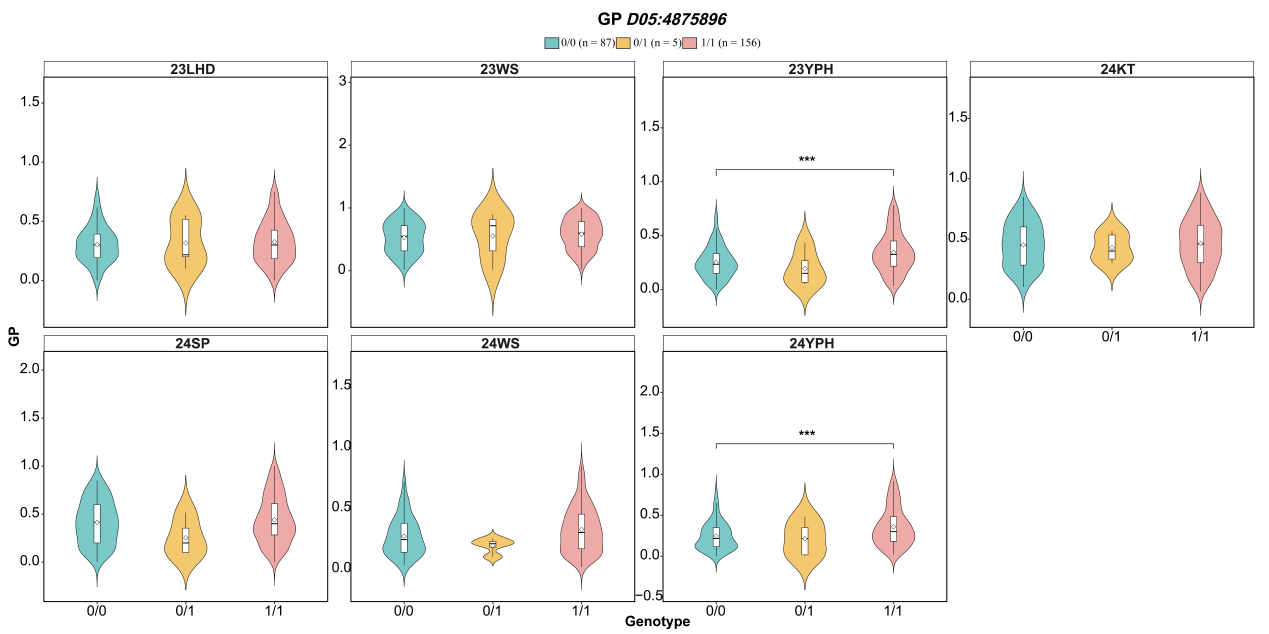

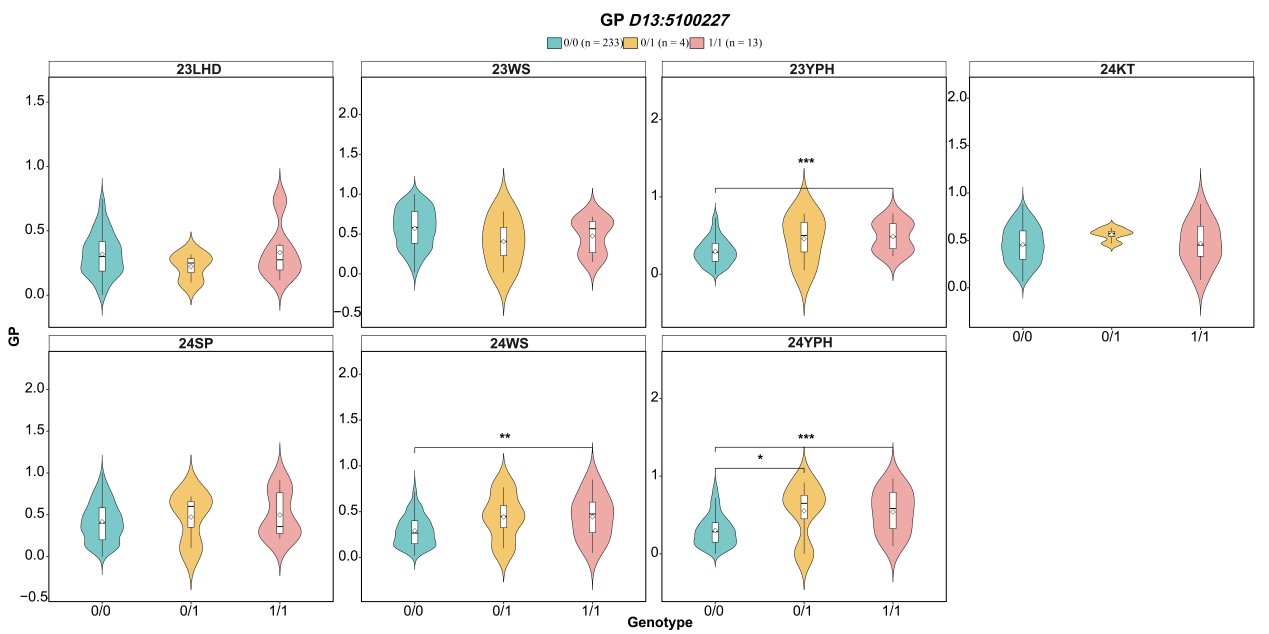

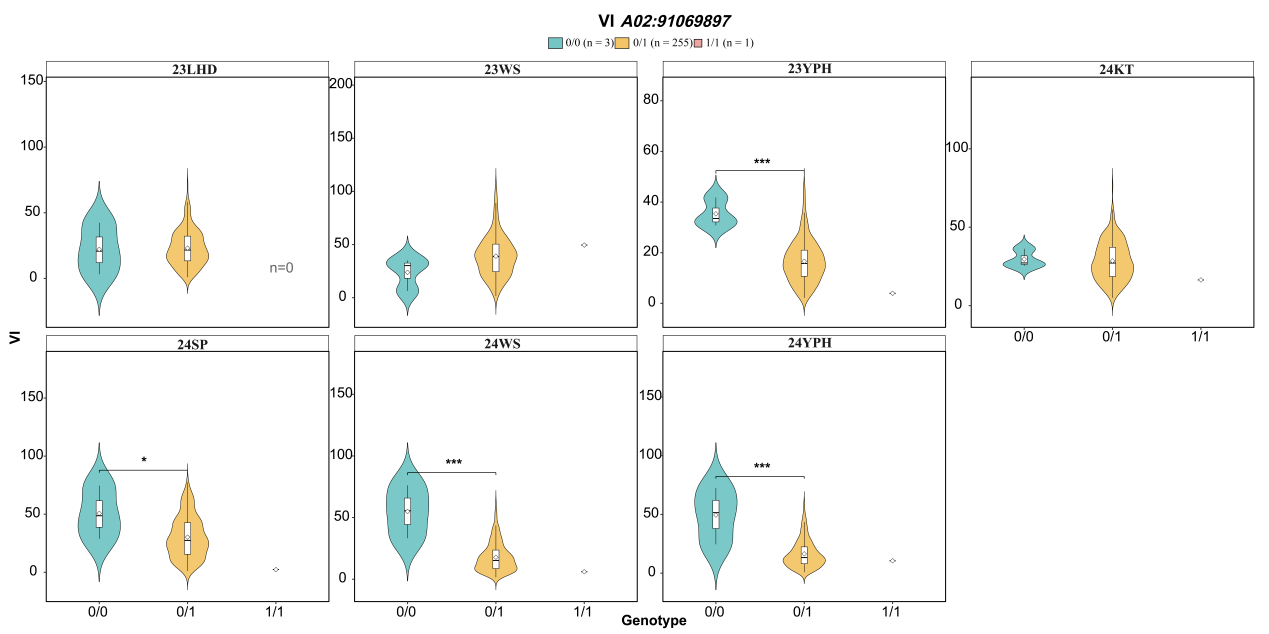

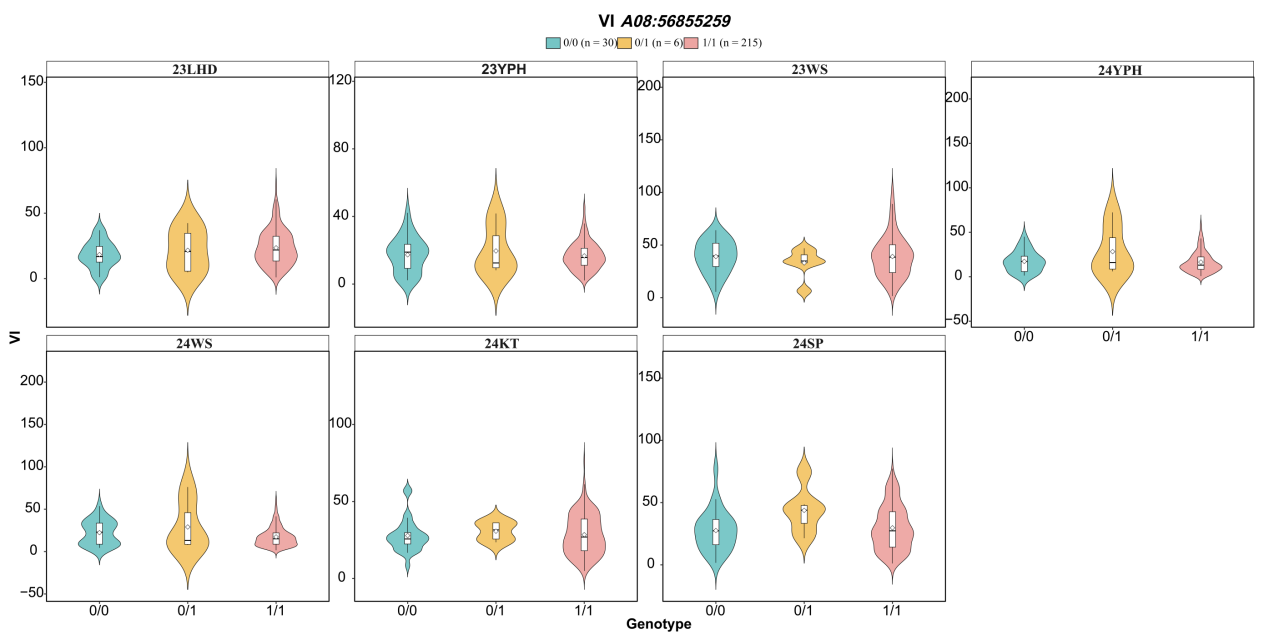

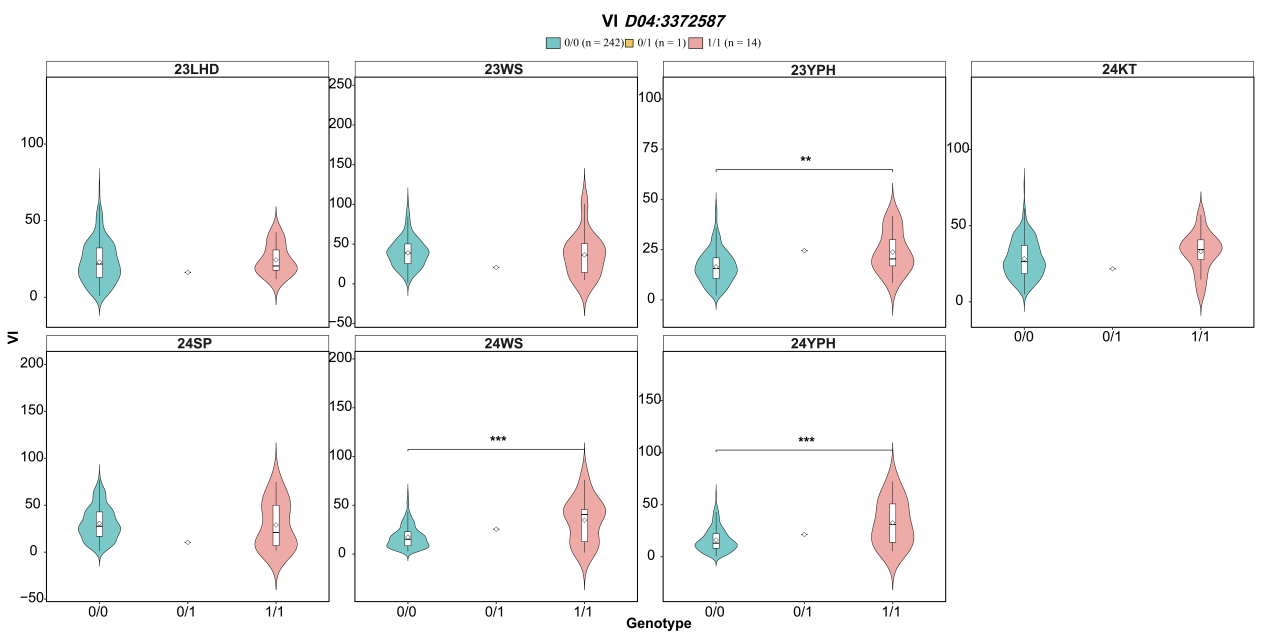

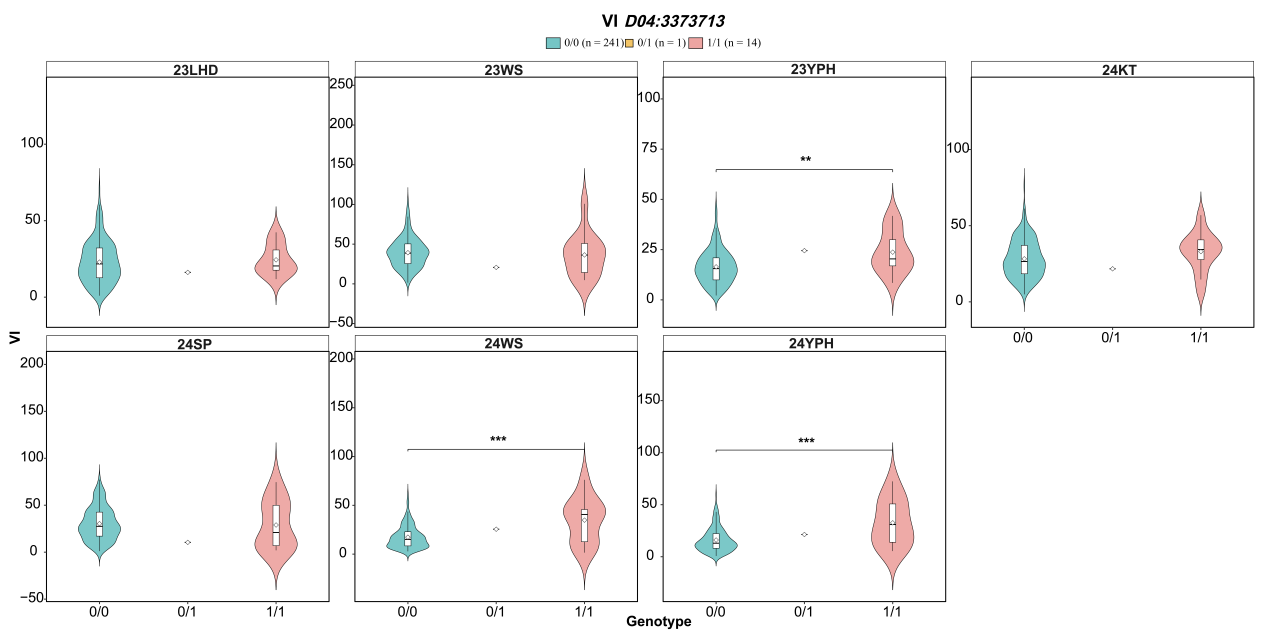



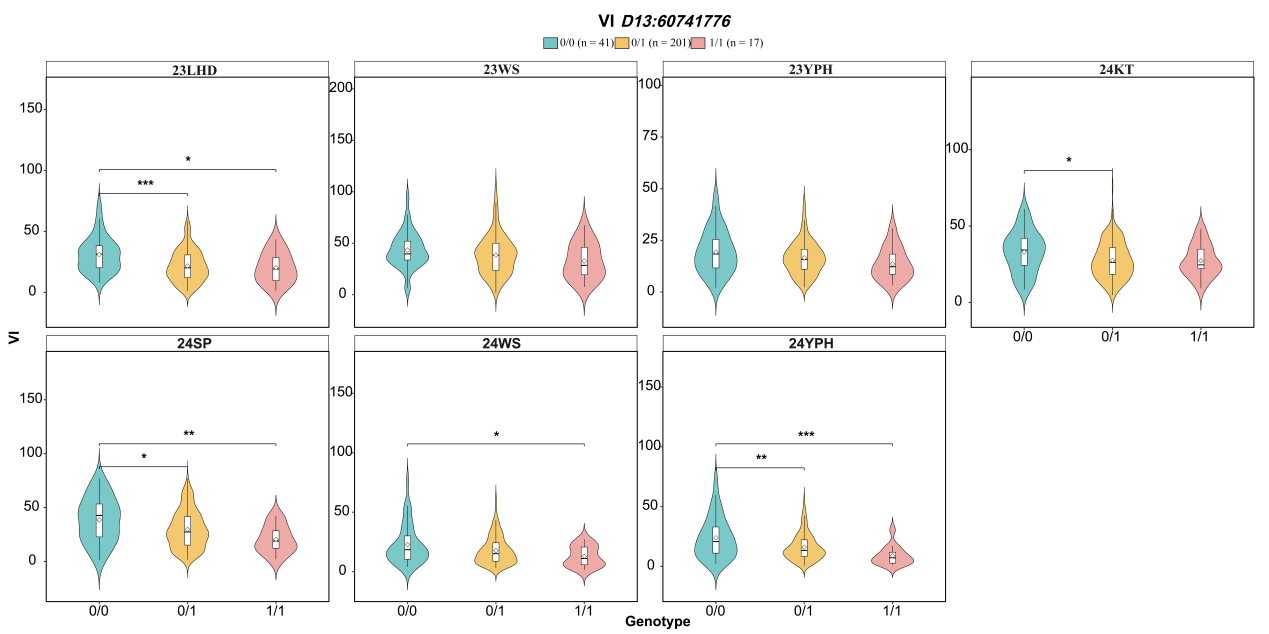


**C**

**B**

**A**

**F**

**E**

**D**

**I**

**H**

**G**

**L**

**K**

**J**

**Supplementary Figure. S8** (A–O) Violin plots illustrating genotype–phenotype analysis of 15 significant SNP loci for three traits.

**O**

**N**

**M**
